# Stress experiences of healthcare assistants in family practice at the onset of the COVID-19 pandemic: a mixed methods study

**DOI:** 10.3389/fpubh.2023.1238144

**Published:** 2023-09-04

**Authors:** Hanna Schrader, Jessica Ruck, Gábor Borgulya, Sandra Parisi, Jana Ehlers-Mondorf, Hanna Kaduszkiewicz, Stefanie Joos, Anna Grau, Klaus Linde, Ildikó Gágyor

**Affiliations:** ^1^Department of General Practice, University Hospital Würzburg, Würzburg, Germany; ^2^Institute of General Practice, Christian-Albrechts University of Kiel, Kiel, Germany; ^3^Institute for General Practice and Interprofessional Care, Tübingen University Hospital, Tübingen, Germany; ^4^Institute of Clinical Epidemiology and Biometry, University of Würzburg, Würzburg, Germany; ^5^Institute of General Practice and Health Services Research, School of Medicine, Technical University Munich, Munich, Germany

**Keywords:** healthcare assistants, COVID-19 pandemic, psychological burden, stress factors, primary care

## Abstract

**Background:**

At the beginning of the pandemic in 2020, healthcare assistants in general practices were confronted with numerous new challenges. The aim of the study was to investigate the stress factors of healthcare assistants in March/April 2020 as well as in the further course of the pandemic in 2020.

**Methods:**

From August to December 2020, 6,300 randomly selected healthcare assistants in four German states were invited to participate in the study. We performed a mixed methods design using semi-structured telephone interviews and a cross-sectional survey with quantitative and open questions. The feeling of psychological burden was assessed on a 6-point likert-scale. We defined stress factors and categorized them in patient, non-patient and organizational stress factors. The results of the three data sets were compared within a triangulation protocol.

**Results:**

One thousand two hundred seventy-four surveys were analyzed and 28 interviews with 34 healthcare assistants were conducted. Of the participants, 29.5% reported experiences of a very high or high feeling of psychological burden in March/April 2020. Worries about the patients’ health and an uncertainty around the new disease were among the patient-related stress factors. Non-patient-related stress factors were problems with the compatibility of work and family, and the fear of infecting relatives with COVID-19. Organizational efforts and dissatisfaction with governmental pandemic management were reported as organizational stress factors. Support from the employer and team cohesion were considered as important resources.

**Discussion:**

It is necessary to reduce stress among healthcare assistants by improving their working conditions and to strengthen their resilience to ensure primary healthcare delivery in future health crises.

## 1. Introduction

The World Health Organization declared the COVID-19 outbreak a pandemic on 11 March 2020, we will refer to this as the “Corona pandemic” hereafter (1). Numerous studies have examined the stress experience of hospital staff during the first wave of the pandemic in March–April 2020 (2–4). In Germany, the majority of COVID-19 patients was treated by primary care physicians (5). Further, healthcare assistants (HCAs) in primary care practices have played a key role in the pandemic management and have contributed significantly to relieving the outpatient sector during the pandemic. HCAs support physicians in the examination and treatment of patients. They are also responsible for patients’ appointment management, organization of practice procedures, performing billing for health insurance services and laboratory activities. For patients, they are often the first point of contact when problems arise. In contrast to healthcare workers in other countries, HCAs have more an assisting and managing function (6, 7). It is well known that health professions are considered to be highly stressful (8) and the pandemic has promoted the emergence of new stressors and the reinforcement of existing stressors. Previous studies reported a great impact of the pandemic on stress experiences, mental health and well-being of healthcare workers by high levels of anxiety, burnout, depression and posttraumatic stress disorder (9). Winefield et al. defined three sources of stress among healthcare professionals: patient-related (e.g., patient care), non-patient-related (e.g., relationships at work, work-life-balance) and organizational sources of stress (e.g., paperwork, support) (10). The sources are related to negative stress experiences, intentions to quit work and negative health effects (8, 10). During the pandemic an acute increase of the already significant shortage of HCAs was reported. According to a survey conducted by the German association of HCAs at the beginning of 2022 among 3,900 HCAs, almost half of them repeatedly considered to give up their profession (11–13). Although the shortage of HCAs is a known health politics problem affecting primary care, their situation has received little scientific attention. While the nursing staff in hospitals often received increased appreciation from the public and policy makers during the pandemic, HCAs in outpatient care were hardly considered (14). Similarly, in ambulatory primary care, the focus of research has generally been the physician sector, while the HCA sector has often been left out completely. International studies looking at the stress experience of primary care health workers showed that they did not feel optimally prepared for the pandemic (15). In this context, it was also reported that primary care nurses felt stressed and overwhelmed. In particular, the lack of protective equipment and the associated anxiety in the workplace were among the stress factors (15, 16). While other healthcare workers were able to work remotely, German HCAs could rarely work from home. Both settings, remote and practice work, showed many stressors and contributed to distress (17). Hence, working in general practices comes along with a risk of infection and the fear of passing the virus to relatives (15, 16). A systematic review identified several occupational risk factors for psychological distress. For example, a high-risk work environment, a lack of specific training and work experiences as well as a lack of social support and stigmatization fostered the development of stress (18). Furthermore, the authors mentioned that resilience, social support and adaptive coping strategies had a protective influence on healthcare workers’ mental health during the COVID-19 pandemic (9) as well as during past infectious disease outbreaks like SARS or MERS (19).

In Germany, other healthcare professions have been focused in research, while HCAs’ stress experiences in pandemic have received little scientific attention. The aim of this study was to explore the occupational stress factors of German HCAs in general practices (GPs) during the initial phase of the pandemic in March/April 2020, when Germany was in lockdown for the first time (20) and during the months afterwards (August–December 2020). Specifically, we aimed to explore the psychological burden of HCAs and to identify patient related, non-patient related and organizational sources of stress. We also wanted to explore HCAs’ individual coping strategies and resources that helped them to deal with the pandemic situation. Our results can support therein to identify approaches to build up resilience of HCAs and find recommendations for policy in the context of future health crises.

## 2. Methods

### 2.1. Study design

**Figure 1:**
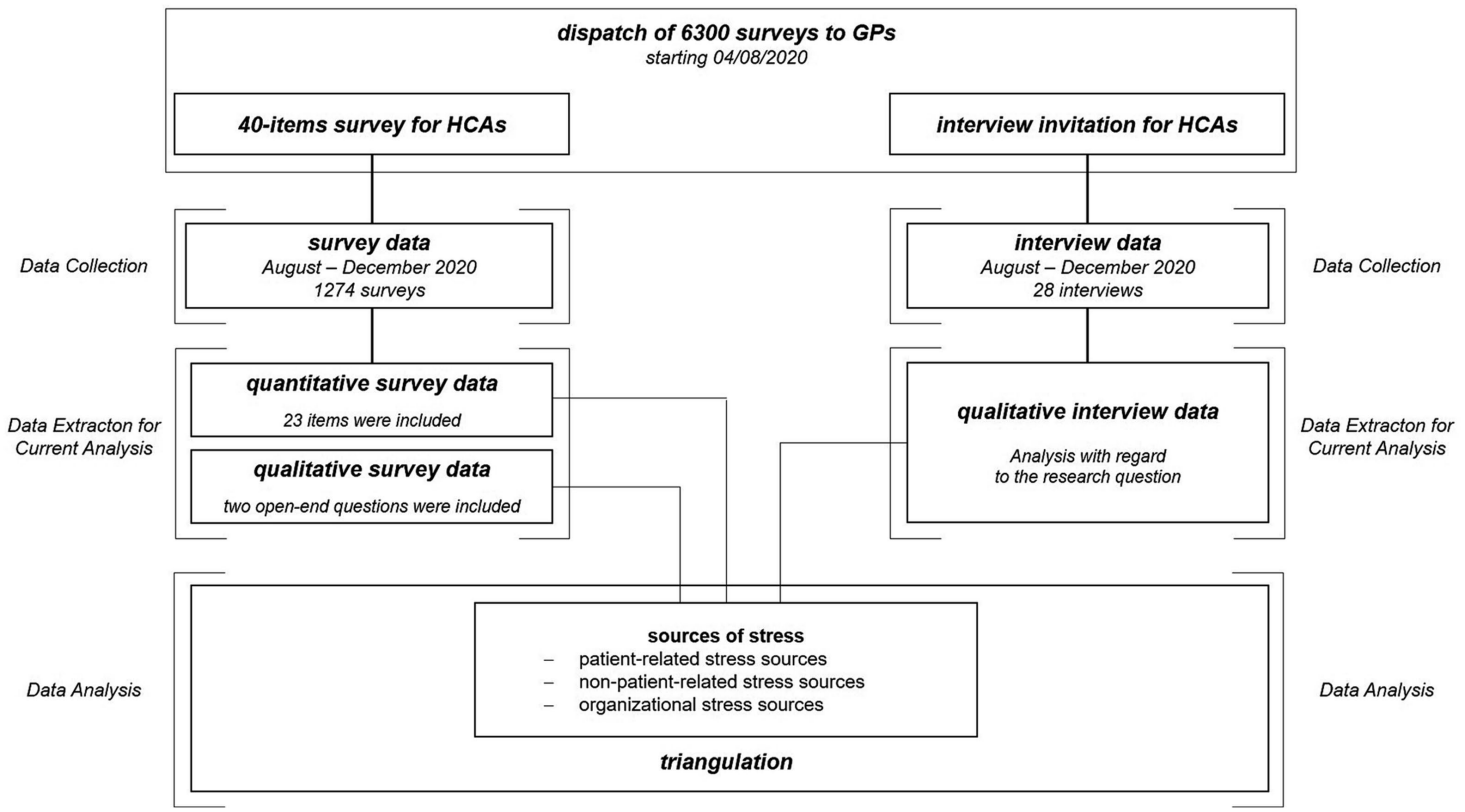
Process of data collection, extraction, analysis and triangulation.

This mixed methods study consisted of a survey, with both structured and open-ended questions, as well as qualitative telephone interviews. We simultaneously collected quantitative and qualitative data from August 2020 until December 2020. While the results of the quantitative and qualitative survey data have not been reported before, the results of the telephone interviews have been published (21). We thus analyzed the qualitative telephone interviews in the sense of the new research question in addition to the survey data aiming to provide a holistic understanding of the burdening experience of HCAs during the pandemic in 2020. We compared the results of all three data sets within a triangulation protocol, using the same methodology as used in a similar study (22). We used methodological data and investigator triangulation to improve the validity of our results (23). An overview of the triangulation process is given in Figure 1.

### 2.2. Study population and recruitment

The study population consisted of 6,300 GPs in four federal states in Germany (for details see Supplementary material S1). When planning the study, incidence rates varied largely, with Southern Germany experiencing far more COVID-19 cases. In order to gain a more holistic understanding of the experiences in general practices we therefore included two federal states in the north (Schleswig-Holstein and Mecklenburg-Western Pomerania) and the south of Germany (Bavaria and Baden-Württemberg). The practices were randomly selected by Arztdata, a commercial provider of address lists (24). We invited 1,980 practices in Schleswig-Holstein and Mecklenburg-Western Pomerania and 4,320 practices in Bavaria and Baden-Württemberg (proportionally to the overall number of GPs within the federal states, for details of the sample size calculation see Supplementary material S2). The survey was conducted from August to December 2020. The first mailing of questionnaires took place on 04/Aug/2020. The practices received two reminders (a first reminder including the survey 6 weeks later, a 2nd reminder of a post card after another 4 weeks). Survey responses have to be assessed in the context of the COVID-19 pandemic course. Supplementary material S3 illustrates the relation between the distribution of responses and infection rates.

From each selected GP one physician and one HCA were eligible to participate in the cross-sectional survey and in the interviews. Results of the study with general practitioners are published elsewhere (25, 26). As already described only five HCAs registered for the telephone interviews in response to the first invitation (21). Therefore, 100 of the 6,300 invited HCAs were randomly selected and invited again by telephone to participate in the interviews. Of these, 23 HCAs participated, 50 declined participation and 27 were not reached. Participation was remunerated with 30€. HCAs who had already participated before were also paid 30€ retroactively (21) (for details about the study recruitment see Supplementary material S1).

### 2.3. Data collection

The research team was multidisciplinary and consisted of practicing clinicians, academic GPs and psychologists (JR, HS, GB, SP, IG, HK, and SJ), quantitative (GB and KL) and qualitative (IG, SP, and HK) research experts and a doctoral student (JE-M). Interviews were conducted by one researcher (JE-M) who was trained and supervised by an experienced qualitative researcher (HK).

#### 2.3.1. Quantitative data: the survey

The 40-items survey (see Supplementary material S4) was developed in a participatory process involving family physicians, junior doctors, psychologists, and other scientific staff from eight Departments of General Practices to ensure the relevance of the questions for the target group. Then, the survey was tested in one of the teaching practices of the Department of General Practice of Würzburg, belonging to the focus group. Data was collected at one measurement time point between August and December 2020. The questionnaire contained retrospective questions about HCAs’ experiences during the initial period of the pandemic in March/April 2020, as well as the current situation at the time of the survey (August to December 2020) and their future expectations. The survey consisted of single-choice and multiple-choice questions with the option “others” offering the possibility of specification or adding additional information, likert-scaled questions and open-end questions. The feeling of psychological burden was assessed on a 6-point likert-scale. A paper-pencil format was used.

#### 2.3.2. Qualitative data: open end survey-questions

We included responses to two open-ended questions of the survey (see Supplementary material S4). One question asked about wishes for other future pandemic waves (question 7: “What measures/offers would you wish for in a case of another pandemic wave?”) and the other question asked about wishes of support (question 16: “Are there other forms of support you would like in a future pandemic wave?”).

#### 2.3.3. Qualitative data: telephone interviews

The main results of the qualitative interview data on experiences with the COVID-19-pandemic as seen from the perspective of German HCAs are already published (21). The semi-structured telephone interviews were conducted between August and December 2020. The interview guide appears in Supplementary material S5. For this study we performed a further analysis of the data with a focus on burdening experiences, stress factors and coping strategies.

### 2.4. Data analysis

Quantitative data were analyzed by GB, while qualitative data were analyzed by HS, JR, and JE-M. We identified burdensome experiences and stress factors in all three datasets and then grouped them using a model of stress sources in healthcare defined by Winefield et al. (10).

#### 2.4.1. Analysis of quantitative survey data

Data entry was performed in Redcap (27). The data was exported and analyzed using SPSS (Version 26, data export function) (28) and Python (version 3.5). Cases with missing values (m) were excluded from the respective analyzes only. For this study we selected 23 survey items based on Winefield’s sources of stress for analyzes and assigned them to the stress-related categories (10) (Supplementary material S6). For the descriptive analyzes, absolute frequencies, minimum and maximum values, means, medians and standard deviations were calculated depending on the nature of the data. Data measured on ordinal scales were compared using the Vargha-Delaney A statistics, implemented (GB) in Python (29). The “A” formula was also used for comparisons of ordinal variables to a reference level (e.g., changes in feeling overburdened over the time) using “no changes” as reference category. We hypothesized that temporal changes may have been present. When the survey included corresponding questions, these hypotheses were tested. *P* values and confidence intervals (95% CI, shown between brackets) for A values were calculated using bootstrap, implemented (GB) in Python (30). In the relative frequency calculations “I do not know” (d), missing (m) and “no need” answers were not included in the denominator.

#### 2.4.2. Analysis of qualitative survey data

Open questions were explored through qualitative methods as described in the section below on telephone interviews. As described previously categories were identified deductively-inductively (31–34). Therefor the main categories were derived by the research objectives and topics to be triangulated, whereas the themes emerging within these categories were derived inductively from the text. Categories were then grouped to the stress factors aligned to Winefield et al. (10).

#### 2.4.3. Analysis of qualitative telephone interview data

All interviews were analyzed again. Aspects related to the burdening experience were extracted from the existing coding framework and analyzed in more depth using MAXQDA software (Vers. 2020). New codes were assigned deductively with regard to the research question and were discussed several times by the research team (HS, JR, and JE-M) until agreement was achieved. Themes were then grouped to the stress factors aligned to Winefield and colleagues (10). Supplementary material S7 shows the published coding framework and the restructured framework focusing on stress experiences.

### 2.5. Triangulation of results

We identified the key findings of each data set and listed them within a triangulation protocol (GB, HS, and JR for quantitative results, JE-M, JR, HS for qualitative results; Supplementary material S8). Key findings were compared and categorized as agreement, partial agreement, silence (e.g., not mentioned due to study design) or disagreement by HS, JR, and JE-M. SP supervised the triangulation process. Differences were resolved by discussion.

### 2.6. Ethics statement

Participation was anonymous as the questionnaires were returned by mail via return envelope without indicating a sender. Informed consent for the telephone interviews to be audio recorded was given by mail and e-mail and was signed by all participants. Details of the interviews which might allow to identify a person were anonymized during the transcription (21).

## 3. Results

### 3.1. Sociodemographic characteristics of the respondents

There were 1,274 surveys available for analysis (response rate 21.1%, details in Supplementary material S1). Most of the HCAs were female [98.9%; missing (*m*) = 17]. The median age was 43 years and their median number of working years in the profession was 20. The type of practice they were working in was a Individual practice for 58.2% of the HCAs and Joint practice for 35.4% (Table 1). For a detailed description of qualitative interview participants see Supplementary material S9.

**Table 1:** Sociodemographic data of survey participants and practice characteristics.

	Mean	Median	Range
Age in years (*N* = 1,175; *m* = 99)	42.5	43	18–76
Years of profession (*N* = 1,161; *m* = 113)	21.4	20	1–57
Number of HCAs (*N* = 1,231; *m* = 43)	4.7	4	1–35
Number of physicians (*N* = 1,232; *m* = 42)	2.1	2	1–16
COVID-19 risk group* (*N* **= 1,187;** ***m*** **= 87)**	**Valid** ***n*** **(%)**		
Yes	210 (17.7%)		
No	977 (82.3%)		
**COVID-19 risk group household (*N*** **= 1,227;** ***m*** **= 47)**	**Valid** ***n*** **(%)**		
Yes	581 (47.4%)		
No	646 (52.6%)		
Position in practice** (*N* **= 1,229;** ***m*** **= 45)**	**Valid** ***n*** **(%)**		
HCA trainee	9 (0.7%)		
Employee HCA	840 (68.3%)		
Senior HCA	347 (28.2%)		
Other**	33 (2.7%)		
Practice structure*** (*N* **= 1,213;** ***m*** **= 61)**	**Valid** ***n*** **(%)**		
Individual practice	741 (58.2%)		
Joint practice	451 (35.4%)		
Medical care center	21 (1.6%)		
Practice with several locations	55 (4.3%)		
Practice with single location	84 (6.6%)		
**Location of the practice (*N*** **= 1,242;** ***m*** **= 32)**	**Valid** ***n*** **(%)**		
Rural (<5,000 inhabitants)	477 (38.4%)		
Small town (5,000-20,000)	407 (32.8%)		
City (>20,000-100,000)	235 (18.9%)		
Large city (>100,000)	112 (9.0%)		
Other	11 (0.9%)		
**Federal state of practice (*N*** **= 1,256;** ***m*** **= 18)**	**Valid** ***n*** **(%)**		
Bavaria	550 (43.8%)		
Baden Wuerttemberg	307 (24.4%)		
Mecklenburg-Western Pomerania	192 (15.3%)		
Schleswig-Holstein	207 (16.5%)		

The missing values (m) were not included in the percentage calculation. *Self-assessment. **Other positions: physicians’ assistant, physicians’ secretary, temporary assistant, diabetes adviser and deputy QM manager, nurs, wife of physician, practice manager, supply assistant and/or non-medical assistant (additional qualification of HCAs). ***Multiple choice possible.

### 3.2. Burdening experience of HCAs

#### 3.2.1. Quantitative survey results

A high and very high feeling of psychological burden in March/April 2020 was reported by 29.5% of the participants. Only a few participants reported that professional psychological support was available for them, whereby 52.6% stated that they felt no need for this. The proportion of those who felt overburdened in daily practice decreased at the time of the survey compared to March/April 2020 (Table 2).

**Table 2:** Psychological burden of HCAs and availability of support.

Psychological burden in March/April 2020 (*N* = 1,247; *m* = 27)		Availability of psychological support (*N* = 1,231; *m* = 43)	
	Valid *n* (%)		Valid *n* (%)
Not at all	194 (15.5%)	Never	489 (83.7%)**
Very little	127 (10.2%)	Rarely	32 (5.5%)
Little	215 (17.2%)	Sometimes	28 (4.8%)
Medium	343 (27.5%)**	Often	13 (2.2%)
High	234 (18.8%)	Always	22 (3.8%)
Very high	134 (10.7%)	No need	647
Feeling of overburden in daily practice (for more details see Supplementary material S9)			
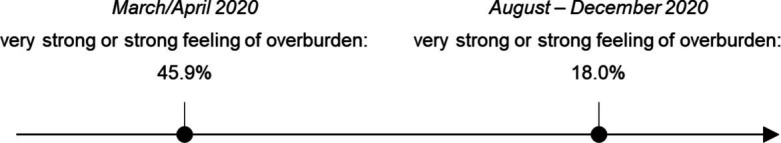			
Vargha-Delaney A [*A* = 34.5% (32–36) *p* < 0.001] [*A* = 26.2% (24–28) *p* < 0.001]			

The missing values (m) and the answer “no need” were not included in the percentage calculation. **The median response is highlighted in bold for scale questions.

#### 3.2.2. Qualitative telephone interviews

Most HCAs reported suffering from experiences of burden at the beginning of the pandemic. They noted an increased petulance, and felt overwhelmed, burned out, and tainted with the situation. Worries about their own health and, even more, the health of their families through infection risk strained the HCAs (VS1). Also, physical illnesses were reported as a result of the high stress level (VS2). A wish for offers of professional psychological counseling was expressed (VS3).

### 3.3. Stress sources of HCAs in the context of the COVID-19 pandemic

In the following section the results are presented thematically triangulated according to the stress sources defined by Winefield and colleagues (10). Exemplary additional verbatim quotes (*VS*) and comments (*VC*) are listed in Supplementary materials S10, S11.

#### 3.3.1. Patients as a source of stress

##### 3.3.1.1. Quantitative survey results

In March/April 2020, 31.7% of the HCAs felt bad and 12.7% very bad about caring for COVID-19 patients. The feeling of being able to take care of COVID-19 patients was substantially improved at the time of the survey. Further worries expressed by the HCAs were getting insufficient or contradicting information about COVID-19 (50.1%) and to overlook COVID-19 among their patients (36.1%). In the opinion of HCAs, in March/April non-COVID-19 patients canceled appointments out of fear (91.2%). 8.7% of HCAs thought that non-COVID-19 patients have been harmed through the pandemic situation in March/April (Table 3).

**Table 3:** Summary of patient-related stress factors.

Survey QUAN	
**Ability to care for COVID-19 patients in March/April 2020 (*N*** **= 1,242;*m*** **= 32)**	**Valid** ***n*** **(%)**
Very poor	158 (12.7%)
Poor	394 (31.7%)
Medium	**387 (31.2%)**
Good	244 (19.6%)
Very good	59 (4.8%)
**Ability to care for COVID-19 patients in Aug-Dec 2020 (*N*** **= 1,239;** ***m*** **= 35)**	**Valid** ***n*** **(%)**
Very poor	25 (2.0%)
Poor	102 (8.2%)
Medium	362 (29.2%)
Good	**598 (48.3%)**
Very good	152 (12.3%)
**Current concerns regarding practice (*N*** = 1,274)**	**Valid** ***n*** **(%)**
Overlooking COVID-19 disease in patients	460 (36.1%)
I am not concerned about COVID-19	132 (10.4%)
Contradictory or too little Information on COVID-19	638 (50.1%)
Patients being infected by the practice team	441 (34.6%)
**Changes in care of non-COVID-19 patients in March/April 2020 (*N*** = 1,274)**	**Valid** ***n*** **(%)**
Patients have canceled appointments out of fear	1,162 (91.2%)
Reduction in unnecessary consultations	868 (68.1%)
Patients have been harmed	111 (8.7%)
No changes	44 (3.5%)
**Survey QUAL**	**Interviews QUAL**
Disagreements with impatient and unfriendly patients Desire for educational work and information Desire for more educational work and information for the population Objective coverage through media for avoiding panic among patients	Dealing with patients’ emotions Disagreements with patients on hygiene measures Increase in telephone calls with unsettled, lonely patients Patients with a cold were afraid and came to exclude Corona Worries about patient’s health Patients avoided the GP because of fear of infection Patients being harmed Death of individuals (a small number of patients) Uncertainty with new disease Poor information about the disease Uncertainty how to deal with infected patients Uncertainty how to deal with SARS-CoV2 tests
Example verbatims: What measures/offers or other forms of support you would like in a future Pandemic wave? VC1: Patients being *“reasonable,” “less stressful,” “patently,” “not so bad”* VC2: *“Sympathy of patients and relatives”* VC3: *“Support from the health departments so that patients can be well informed and do not have to turn to us helplessly because the departments are overloaded.”* VC4: *“Structured information material for patients”* VC5: *“Communication of the real risk of disease. Avoidance of panic and horror messages. Information on health protection measures.”*	Example verbatims VS9: *“[…] Also because many patients are more dissatisfied, more aggressive, which I already said, you have to be scolded why things are not moving forward. People have to stand outside in the rain in the cold. But as I said, they are all just people and we cannot do more than work. Of course, we also have patients who really praise us and say: “Wow, that’s great how you do everything here and how you handle it. You have both encouragement, but also patients who are sometimes, I would say, indignant.” (No. 7, pos. 12)* VS8: *“That’s more now, as the numbers are getting higher now, of course we are getting more panicky patients, need to make more phone calls because patients just panic and of course they call us, and we have to reassure them, explain a little bit and they were, are now also sometimes afraid to come to the surgery.* “*(No. 20, pos. 16)* VS4: *“Now what to speak, what is a worried patient and what is a sick patient, who really needs help?” (No. 2, pos 8)*

The missing values (m) were not included in the percentage calculation. *The median response is highlighted in bold for scale questions. **Multiple choice possible.

##### 3.3.1.2. Qualitative survey results

HCAs reported difficulties with stressful, unfriendly and impatient patients (VC1, VC2, and VC3). There was a wish for more educational work for the population and that media coverage should be more objective to avoid causing panic among patients (VC3, VC4, and VC5).

##### 3.3.1.3. Qualitative telephone interviews

Some participants reported uncertainty in the team due to the new disease pattern (VS4). There was uncertainty about how to deal with infectious patients and SARS-CoV-2 tests (VS5). As also described previously, poor information about the new virus and how to get protected from it contributed to the stressful experience (VS6) (21). Some HCAs reported disagreements with patients regarding the hygiene measures (VS7) and an increase in telephone calls with unsettled and lonely patients (VS8). There were experiences with aggressive as well as thankful patients (VS9). Some HCAs worried that patients would be harmed because they did not go to the doctor because of fear of COVID-19 (VS10) and even dreaded deaths in a small number of patients (VS11).

#### 3.3.2. Non-patient sources of stress

##### 3.3.2.1. Quantitative survey results

The fear that family members could become severely ill with COVID-19 was greater than the fear of own illness. For 27.3% of the HCAs the pandemic caused difficulties in reconciling work and family life (e.g., unavailable childcare), 15.5% only sometimes had such difficulties (Table 4). Other non-patient sources of stress were worries that colleagues would get infected or that the team would infect patients and concerns of suffering financial damages. Some HCAs (6.2%) were worried about losing their jobs (Supplementary material S12).

**Table 4:** Summary of non-patient-related stress factors.

Survey QUAN	
**Afraid of getting sick with COVID-19 (*N*** **= 1,254;*m*** = 20)**	**Valid** ***n*** (%)*
Agree completely	196 (15.6%)
Rather agree	289 (23.0%)
Indifferent	**224 (17.9%)**
Rather disagree	375 (29.9%)
Disagree completely	170 (13.6%)
Afraid that relatives could get severely sick with COVID-19 (*N* = 1,254; *m* = 20)**	Valid *n* (%)*
Agree completely	477 (38.0%)
Rather agree	**414 (33.0%)**
Indifferent	143 (11.4%)
Rather disagree	155 (12.4%)
Disagree completely	65 (5.2%)
**Difficulties in balancing family and career (*N* = 1,249; *m* = 25)**	Valid *n* (%)*
Yes	342 (27.4%)
Sometimes	194 (15.5%)
No	713 (57.1%)
**Survey QUAL**	**Interviews QUAL**
Desire to protect from COVID-19 Removing infectious patients to protect other patients Fear of infect their relatives with COVID-19 Complaining about no regular testing of medical personnel to protect relatives Wish for support in child care	Decrease in quality of healthcare Reduced services (e.g., ecg) because of hygiene measures Closed GP completely Sick leaves of HCAs to protect themselves and their relatives Feeling less resilient because of pandemic measures Contact restrictions Restrictions on leisure Discussions about measures in private environment Stigmatization in private environment Pro and cons of reduced working hours Financial problems More leisure time Struggle because of lack or limited child care Problems in handling job and family Quitting job as last resort More work for colleagues because of staff shortage Feeling strengthened through improved team work due to new challenges
Example Verbatims: What measures/offers or other forms of support you would like in a future Pandemic wave? VC6: *“Reopening of the test station by the coordinating doctor to avoid contact between infected or suspicious patients and non-infected patients.”* VC7: *“keep COVID-19 out of practices as much as possible”* VC8: *“That medical staff are also tested. Medical practices are left alone with this.”*	Example verbatims VS12: *“And yet when I think that some now here in the area, yes some have closed, actually closed their surgeries, out of concerns about the Corona. “(No. 2, pos. 44)* VS17: “*You cannot go to the beach anymore, you cannot do sports, you cannot go for walks by the sea, now you have to go into the forest […]. So you have to change. But that does not mean / you have to change, but you are already more organized with the meetings of friends, they are phoned. So it works differently.” (No. 19, pos. 97)* VS24: *“Well, yes, I also have a small child who is in the kindergarten. Then the time when the kindergartens were closed was also a huge drama at the beginning. Of course, I was always afraid at the beginning that I would spread something to my daughter or to my grandmother, who is actually very ill. But at the beginning, in March and April, contacts were limited and it was difficult to manage child and work, because at the beginning it wasn’t under emergency care.” (No. 25, pos. 38)* VS27: *“For a short time it was quite difficult. I have to be honest that every one of us reached the limit. Thank God, we have a great team, where almost no one was sick because they were overworked, or thank God, they did not get sick, good luck. But of course we were often nagging and grumbling at each other, and we were right, because we had to remember that there was a lot of pressure, a lot of responsibility on us.” (No. 25, pos. 16)*

The missing values (m) were not included in the percentage calculation. *The median response is highlighted in bold for scale questions. **For more data see Supplementary material S16.

##### 3.3.2.2. Qualitative survey results

There was the wish for regular testing of medical personnel to protect the relatives (VC8). Working on the frontline, HCAs wanted financial resources for further staff, salary increases and bonus payments (VC6). Furthermore, they wished for better support with childcare.

##### 3.3.2.3. Qualitative telephone interviews

Some practices reduced their treatment services (21) due to fear of infections or had to close completely (VS12, VS13). Many HCAs were worried about infecting family relatives (VS14). In some cases, HCAs took sick leave to avoid infecting themselves or their family (VS15). Contact restrictions led to social isolation (VS16) as well as restrictions on leisure activities (VS17), so that the restrictions had an impact on the mood (VS18). HCAs also reported stigmatization and discussions in their private environment about pandemic measures (VS19, VS20). HCAs occasionally mentioned financial problems due to reduced weekly working hours (V21). Some HCAs experienced the limited childcare at the beginning of the COVID-19 pandemic as very challenging (VS22, VS23). Other HCAs found it very stressful that colleagues were absent due to the lack of childcare (VS24). Individual HCAs also stated that they saw quitting their jobs as a last resort to solve the childcare problem (VS25). Good-willing employers dedicated colleagues and also well-organized schools and teachers were described as helpful (VS26). The good teamwork helped to overcome the challenges (VS27).

#### 3.3.3. Organizational sources of stress

##### 3.3.3.1. Quantitative survey results

In March/April 2020 HCAs spent more time on organizational tasks than before [A = 76% (75–78) *p* < 0.001; Table 5]. At the time of the survey (between August and December 2020) HCAs estimated that they spent less time with organizational duties than in March/April, but still more than before the pandemic. For details about working hours and time spent with patients see Supplementary material S13. Most of the HCAs felt well supported by their employers. They were also satisfied with the actions of their employers (Supplementary material S14) but less [A = 20.1% (18–20) *p* < 0.001] satisfied with the actions of the provincial government (Table 5). FFP2 masks were scarcely available in March/April 2020 (Supplementary material S15).

**Table 5:** Summary of organizational stress factors.

Survey QUAN	
**Impact of the pandemic on organizational activities in March/April 2020**	Valid *n* (%)*
More time than usual	**727 (57.1%)**
Less time than usual	59 (4.6%)
No impact	38.3%
Feeling supported by your employer (*N* = 1,242; *m* = 32)**	Valid *n* (%)*
Very good	599 (48.2%)
Good	**438 (35.3%)**
Medium	150 (12.1%)
Poor	42 (3.4%)
Very poor	13 (1.0%)
**Satisfaction with your state governments handling (*N* = 1,249; *m* = 25)**	Valid *n* (%)*
Very satisfied	76 (6.1%)
Satisfied	367 (29.4%)
Medium	**512 (41.0%)**
Dissatisfied	220 (17.6%)
Very dissatisfied	74 (5.9%)
**Survey QUAL**	**Interviews QUAL**
Wish for personal protection equipment (PPE) Not enough and insufficient PPE Enormous price increases due to higher demand desire for control and order Information management: information should be more objective, uniform, non-contradictory Pandemic measures: more transparent, implementable and should not change permanently Information and latest changes of measures should reach medical professionals before public Frustration through bureaucracy Wish for less bureaucracy and administrative work (especially billing procedure) Wish for more digitalization Wish for support for GPs External structures (e.g., corona medical centers, infection practices) for relief of GP and reducing infection risk in GP Clear responsibility and reliable contact persons (e.g., Department of Health) Seeking for recognition and appreciation Missing recognition and appreciation from the government, media, public More respect for the job of a HCA Equal treatment to other (health) professions	Personal protection equipment (PPE) caused Restrictions in daily work Not enough and insufficient PPE Feeling restricted in work by wearing mouth-nose protection masks (e.g., breathing) Desire for control and order Permanent changing measures Wishes for increased controls on hygiene measures and quarantine rules Wish for harsher punishment for rule violations Feeling overwhelmed through bureaucracy Increased workload due to administration (especially billing procedure) Organization of catch-up appointments for canceled appointments in the 1st lockdown Feeling left on their own and seeking for relief Insufficient support by politicians and public health services Saw themselves left on their own Lack of contact persons Well-organized external structures (e.g., corona centers or separate test practices) for relief of GP and reducing infection risk in GP Offers to talk about worries Lack of appreciation from politicians and the population
**Survey QUAL**	**Interviews QUAL**
Example Verbatims: What measures/offers or other forms of support you would like in a future Pandemic wave? VC9: *“We currently buy gloves, masks* etc. *at far overpriced prices (3–4 times the normal price) in order to protect ourselves.”* VC10: *“More information, we felt very uninformed and helpless at the beginning. We only had information from the news and were supposed to calm patients down. We were told it was our own fault if we did not have protective equipment in stock.”* VC11: *“Clear information and uniform, well thought-out regulations that are easy to implement for a practice.”* VC12: *“Better information on the “bureaucratic aspects,” information sheets on billing procedure and coding were often incomplete, ambiguous, not pertinent for GPs; frequent changes [of information] and we had to tediously collect information on our own.”* VC13: *“That we are given the same attention and support as was given to the hospital staff. After all, GPs are the first point of contact for infectious patients or the fears and worries associated with the pandemic.”* VC14: *“Financial compensation for HCAs! We are also system relevant and not mentioned anywhere!”*	Example Verbatims VS11: *“[…] we had no protective clothing available, we had no FFP2 masks. We actually had nothing at all. “(No. 21, pos. 4)* VS34: *“Well, I have to say that the time was really stressful for me and also, as I said, something changed every day, every day there was another letter from the KV [german: Kassenärztliche Vereinigung; Association of Statutory Health Insurance Physicians] where you had to reorganize yourself again. So we did so much organizational work. […]” (No. 1, pos. 16)* VS22: *“Health departments were not available. We were always told, “Busy,” or, “Contact your family doctor.” We felt downright left all alone. “(No. 16, pos. 4)* *VS35: “It’s kind of annoying because you do not get much support from the departments and government agencies.” (No. 7, pos. 2)*

The missing values (m) were not included in the percentage calculation. *The median levels of ordinal and continuous scale variables are highlighted in outlined numbers. **For more data see Supplementary materials S14.

##### 3.3.3.2. Qualitative survey results

According to the HCAs, sufficient personal protective equipment should be available and affordable in future waves (VC9, VC10). HCAs also wished a better governmental pandemic management (VC10, VC11), less bureaucracy, more digitalization and clear responsibilities, as well as reliable contact persons (VC12). Wishes for future waves of the pandemic were more support for GPs, e.g., by specialized COVID-19 services (corona medical centers, infection practices) (VC14), as well as more support from the Department of Health. According to HCAs the outpatient care was disregarded in medial pandemic reports. HCAs wished more recognition, appreciation and respect for their work and equal treatment to other health professions (VC13).

##### 3.3.3.3. Qualitative telephone interviews

The HCAs reported an increased workload due to administrative duties (VS28) and catch-up dates for canceled appointments during the 1st lockdown in March/April 2020. Another stress factor described by almost all HCAs was the insufficient supply of protective equipment at the beginning of the pandemic, which hindered the daily work (VS29, VS30, VS31). On the other hand, some participants reported feeling restricted in their work by wearing mouth-nose protection masks (e.g., lack of facial expression, headaches, fatigue, breathing problems) (VS32), even if there was a protective function (VS33). HCAs reported that, due to organizational restructuring, measures had to be revised constantly (VS34). During the early pandemic, HCAs felt insufficiently supported and appreciated by policy makers and public health services (VS35, VS36) and saw themselves left on their own due to a lack of contact persons (VS37, *VS* 38). There was a wish for increased controls on compliance with the hygiene measures and quarantine rules, as well as harsher punishment for rule violations (VS39).

#### 3.3.4. Coping strategies

In the interviews, HCAs mentioned numerous strategies to cope with increased burden (e.g., planning day trips instead of vacations) (VS40). Furthermore, the HCAs said they had actively strengthened their ties to family and friends (VS41). Some participants reported that having a positive attitude, accepting and allowing negative feelings had helped them to cope with the pandemic (VS42). Some HCAs reported that they also had done extensive private research on COVID-19, which had contributed to a sense of security among them (VS43) (Supplementary material S17).

### 3.4. Triangulation of the results

The key findings (*N* = 33) across all data sources are described in the triangulation protocol (Supplementary material S8). Allover, there were eight agreements between the three data sources. There was a high number of agreements between qualitative survey data and qualitative interviews (25 agreements, 8 silences), whereas there was a high number of silence between quantitative and qualitative survey data, indicating a different focus of questions and that participants used the qualitative questions to complement the information provided within quantitative variables. There was no disagreement.

## 4. Discussion

### 4.1. Summary of the main findings

Our results show a high psychological distress of the HCAs at the beginning of the pandemic, which caused negative feelings such as anger and frustration. Patient-related sources of stress during the pandemic were for example non-COVID-19 patients being harmed and an uncertainty in patient care due to a lack of experience with COVID-19. Non-patient-related stress factors were compatibility problems of work and family, as well as the fear of HCAs infecting their relatives with COVID-19. Organizational sources of stress were a lack of availability of protective equipment and an increase in organizational and administrative workload. Furthermore, the HCAs complained about a lack of appreciation and support from policy makers. HCAs used problem-focused strategies (e.g., implementation of hygiene measurements in a creative way), emotional-focused strategies (e.g., leisure time, social resources) and attributional-focused strategies (e.g., optimism, reframing) for coping stress. Professional psychological support, on the other hand, was considered hardly available. Triangulation of results showed agreement and silence between key findings, indicating that participants often used qualitative questions to complement the information provided within the quantitative survey related to their experience of stress.

### 4.2. Comparison with existing literature

In numerous countries, high stress levels in healthcare workers were reported, especially at the beginning of the pandemic (3, 4, 35, 36). Quantitative studies identified an increased incidence of mental and psychosomatic illness among healthcare workers (37, 38). Consistent with these findings, our data reveal high level of stress and described psychological burden as well as psychosomatic illnesses in the context of the pandemic, which were considered as stress-related. Further, changes in the relationship to patients through conflicts with regard to hygiene measures were identified as a source of stress, which corresponds to literature (39–41). Reviews from Zhang et al. and Rossi et al. revealed an increase of pandemic related workplace violence (42, 43). While our study did not report instances of physical aggression, HCAs considered the increase of verbal violence of patients against them as stressful. Non-patient sources of stress were a lack of childcare and fear of passing the virus to family members as also seen by Ashley et al., Robinson et al., and Frenkel et al. (16, 44, 45). The fear of infecting family members and friends was greater than the fear of HCAs’ own infection, indicating a high feeling of responsibility as also described among general practitioners (46). Furthermore, few cases in the study also reported the stigmatization of HCAs during the pandemic as reported in other study results (47). In other countries and also in our results, an unstructured flow of information was mentioned as an organizational source of stress in everyday practice (18, 48). In general, stress arises if external or internal demands perceive as threatening and unable to cope (49). This could be also observed in our data. In March/April 2020, the HCAs were concerned to get too little and unstructured information about the unknown disease and felt not good prepared to care for COVID-19 patients. They reported uncertainty and constantly changing conditions that made it hard to feel able to cope with the pandemic situation and may frustrated the need for control and orientation (50). The months afterwards, the caring abilities increased and the feeling of overwhelm decreased showing a successful adaption of HCAs to the pandemic situation. A change in weekly working hours was particularly evident at the onset of the pandemic, with a greater amount of organizational activities and less time spent with patients (51). HCAs expressed frustration about policymakers’ lack of appreciation for their work (52). As also reported by the media, for the future the participants wished to be considered for bonus payments like other health care staff, as well as for adjustment of their salaries in view of the increased workload (53). A relief of the GPs by public health services and also more structured information flow was considered essential, which is in line with the results of other studies (48, 54, 55). The literature emphasized the relevance of coping abilities and resilience of healthcare workers during crises (18, 56). The HCAs reported different strategies to cope with new pandemic challenges and the increased stress. As a problem-focused coping strategy, practice teams often resorted to creative solutions in the face of problems such as a lack of protective equipment or difficulties in implementing hygiene concepts. Working as a team strengthened cohesion (54, 57–59). Social resources like family and friends found to be supportive which is in line with literature mentioned social support as a protective factor of stress (18, 60). Optimism and reappraisal of the pandemic situation helped some HCAs as a cognitive coping strategy (18).

### 4.3. Strengths and limitations

This is, to our knowledge, one of the first mixed methods studies using survey data and qualitative interviews to explore the burdening experience of German HCAs within primary healthcare in relation to the COVID-19 pandemic. The study included a relatively large randomly selected sample. Nevertheless, with a response rate of 21%, it is unclear whether the results are representative of HCAs in Germany. A similar response rate was found in the survey of physicians (26). This could indicate an influence of physicians’ participation to the participation of HCAs as a selection bias. The study design (cross-sectional) does not allow a detailed assessment of the impact of the pandemic on HCAs. Although survey questions assessed different time points [March/April 2020 and “current” (corresponding between August and December 2020)], the single-stage survey allows little inference about the dynamics of the pandemic and the experiences of HCAs in the course of it. In addition, individual participant responses may have been affected by memory lapses (recall bias). An additional limitation is the lack of standardized measurements of psychological burden (e.g., for anxiety and depression) as well as stress experiences. Further bias may have occurred in the recruitment of HCAs for the qualitative interviews, with a subsequent introduction of an incentive due to recruitment difficulties. In addition, some HCAs conducted the interviews on the premises of the practice while others off-site, which may have led to a bias in the response pattern, particularly regarding team dynamics and employers. Due to the long study period of 5 months, systematic biases in the response tendencies could have arisen as a result of the pandemic dynamics both in qualitative and quantitative data. The study was conducted during a period when the number of infections in Germany was comparatively low, which might have influenced the differences we detected with regards to the burdening experience between the two time points we assessed in our survey (March/April 2020 and “current”). Even if the pandemic in March/April 2020 represented a previously unknown exceptional situation, GP teams were repeatedly confronted with new stress situations during the course of the pandemic, which represent an extreme intervention in the everyday practice (e.g., vaccination campaigns) (13, 61). Thus, it seems likely that the results of this study will be transferable to future pandemic events. Furthermore, our study has investigated an underrecognized study population that needs more attention in further research.

### 4.4. Implications

The COVID-19 pandemic was a dynamic infection event, whereby it can be assumed that in the long term further pandemics will lead to changes in the daily routine of GPs (62–64). This study can therefore help to better understand the stressful and supportive factors of HCAs as an occupational group that has received little attention in research to date. Our results show the important role of the public health sector today and in the future in terms of ensuring the productivity and well-being of HCAs in the pandemic. Future research topics should therefore include how to improve the collaboration of GP teams with employees of the public health department. Regarding to HCAs’ increased burden due to the pandemic expounded by our study, services should also be created for the outpatient sector that can help deal with workplace-related stress. To deal with future challenges, the resilience of HCAs should be promoted and strengthened (65). This can help prevent overwork and ensure an effective, adaptable and sustainable work team. The aforementioned sources of stress, such as challenging patients, organizational factors and regulations, can provide starting points for this. Especially in view of the shortage of HCAs not only in Germany, it seems fundamental to improve their working conditions in order to be able to ensure primary health care delivery (66–68). This could prevent HCAs exodus toward other professions not only with regard to future pandemics.

## Data availability statement

The datasets presented in this article are not readily available because due to restrictions within the ethical approval, the datasets cannot be made publicly available. Specific data can be shared upon reasonable request. Requests should be directed to the corresponding authors. Requests to access the datasets should be directed to schrader_h@ukw.de.

## Ethics statement

The studies involving humans were approved by Ethics committee of the University Hospital Würzburg (No. 135/20-am) and the Medical Faculty of the University of Kiel (D 295/20). The studies were conducted in accordance with the local legislation and institutional requirements. The participants provided their written informed consent to participate in this study.

## Author contributions

IG, SJ, HK, HS, SP, and KL conceptualized the initial study. HS and JE-M were involved in study recruitment. JE-M and HK conducted qualitative interviews and transcribed the narratives. GB performed the statistical analysis. HS, JR, JE-M, GB, and SP conducted qualitative analysis and triangulation. IG supervised all processes. HS and JR drafted the initial manuscript. All authors contributed during the process of reviewing and adapting the manuscript and approved the current version for submission.

## Funding

In Baden-Württemberg the project was funded by the Ministry of Science, Research and Arts of the State of Baden-Württemberg. This publication was supported by the Open Access Publication Fund of the University of Wuerzburg.

## Conflict of interest

The authors declare that the research was conducted in the absence of any commercial or financial relationships that could be construed as a potential conflict of interest.

## Publisher’s note

All claims expressed in this article are solely those of the authors and do not necessarily represent those of their affiliated organizations, or those of the publisher, the editors and the reviewers. Any product that may be evaluated in this article, or claim that may be made by its manufacturer, is not guaranteed or endorsed by the publisher.
