# Supplementary material for: Stress experiences of healthcare assistants in family practice at the onset of the COVID-19 pandemic: a mixed methods study
Source: Front Public Health. 2023 Sep 4;11:1238144. doi: 10.3389/fpubh.2023.1238144 (PMC10507357; doi:10.3389/fpubh.2023.1238144)
Supplement: Supplementary file 1 [file Data_Sheet_1.docx]

Supplementary Material

Supplementary material 1:

Supplementary Figure 1. Study recruitment.

Supplementary material 2: Calculation of the sample size of the cross-sectional study.

Biometric report by the Institute for Clinical Epidemiology and Biometry, Julius-Maximilians-University in Wuerzburg, Germany: There are a total of approx. 18691 general practitioners registered with the relevant associations of statutory health insurance physicians, of which 15742 are distributed between Bavaria and Baden-Württemberg in Southern Germany and 2949 between Schleswig-Holstein and Mecklenburg-Western Pomerania in the North. The primary objective is to estimate the proportion of primary care physicians as well as the proportion of health care assistants who reported their excessive workload at the beginning of the Corona crisis with regard to practice activities as high or very high. In the literature, a proportion of approximately 33% (1) has been reported in a similar setting. In this project the proportion is assumed to be 1/3 in each case according to clinical assessment, i.e. about 33%. For feasibility reasons, a total of 6300 GP practices can be contacted, of which 1980 in the North and 4320 in the South. The participation of one physician and one health care assistant per practice is planned. It is expected that 40% of the physicians and 25% of the health care assistants will participate. The resulting sample sizes will be approximately 792 physicians and approximately 495 HCAs in the North and 1728 physicians and 1080 HCAs in the South. To calculate a 95% confidence interval using the score (Wilson) method with a width of 0.065 a case number of 792 is sufficient for physicians in the North and a width of 0.083 is a case number of 495 among health care assistants is sufficient to estimate the expected proportion of 0.33. For the South, 1728 physicians give a width of 0.044 and 1080 health care assistants give a width of 0.056.

Supplementary material 3:

**Supplementary Figure 2.** Timeline of data collection in relation to COVID-19 pandemic in 2020. The black bar chart shows distribution of the survey return dates, the black line shows temporal distribution of infections (proportions of the charts do not correspond). Modified after Schilling et al. (2).

Supplementary material 4: The survey.

| **Date: _ _ . _ _ . 2020** |
| --- |

| 2. How good did you feel caring for patients with covid-19 in **March/April**? | | | | |
| --- | --- | --- | --- | --- |
| very good | good | medium | bad | very bad |
| □ | □ | □ | □ | □ |

| 3. How good do you **currently** feel caring for patients with covid-19? | | | | |
| --- | --- | --- | --- | --- |
| very good | good | medium | bad | very bad |
| □ | □ | □ | □ | □ |

| 4. How has the care of your **non**-covid-19 patients changed due to the pandemic situation in March/April? **(multiple choice, please tick all that apply)** | |
| --- | --- |
| □ patients cancelled appointments because they were afraid | □ patients suffered damages |
| □ reduction of unnecessary consultations | □ no changes |
| □ other (please specify): | |

| 5. What is the **current** patient volume in your practice? | |
| --- | --- |
| □ comparable to the volume before the Pandemic began | □ higher than the volume before the Pandemic began |
| □ lower than the volume before the Pandemic began | □ I don’t know |

| 6. Please rate the following measures in relation to the covid-19 Pandemic:  **(multiple choice, please tick all that apply)** | | | | |
| --- | --- | --- | --- | --- |
|  | we performed that in **March/April** | we are performing that **currently** | we will perform that **in the future** | we do **not** perform that in our surgery |
| (1) Swabs for the detection of SARS-CoV-2 | □ | □ | □ | □ |
| (2) infection consultating hours | □ | □ | □ | □ |
| (3) video consultation | □ | □ | □ | □ |
| (4) Forming special teams in the practice (e.g. multiple shifts) | □ | □ | □ | □ |
| (5) home visits | □ | □ | □ | □ |
| (6) care home visits | □ | □ | □ | □ |
| (7) placing up-to-date information on the  surgery homepage | □ | □ | □ | □ |
| (8) other (please specify) | □ | □ | □ | □ |

| 7. What measures/offers would you wish for **in a case of another Pandemic wave**? |
| --- |
| free text: |

| 8. What are you **currently** worried about in relation to the surgery?  **(Multiple selection, please tick all that apply)** | | |
| --- | --- | --- |
| □ contradictory or too little information about covid-19 | □ that employees could become infected | □ that the practice team could infect patients |
| □ overlooking a covid-19 disease in patients | □ suffering financial losses | □ I have no worries about covid-19 |
| other (please specify): | | |

| 9. How strong was/is your feeling of overburden in view of the Corona Pandemic with regard to your activities in the surgery? | | | | | | |
| --- | --- | --- | --- | --- | --- | --- |
|  | none | very weak | weak | medium | strong | very strong |
| **a) in March/April** | □ | □ | □ | □ | □ | □ |
| **b) currently** | □ | □ | □ | □ | □ | □ |

| 10. How well supported do you feel by your employer with regard to the Corona Pandemic? | | | | |
| --- | --- | --- | --- | --- |
| very well | well | medium | poorly | very poorly |
| □ | □ | □ | □ | □ |

| 11. How satisfied are you with the actions of your government of the federal state in the Corona Pandemic? | | | | |
| --- | --- | --- | --- | --- |
| very satisfied | satisfied | medium | dissatisfied | very dissatisfied |
| □ | □ | □ | □ | □ |

| 12. How satisfied are you with the actions of your employer in the Corona Pandemic? | | | | |
| --- | --- | --- | --- | --- |
| very satisfied | satisfied | medium | dissatisfied | very dissatisfied |
| □ | □ | □ | □ | □ |

| 13. Please answer the following questions about your own personal protection equipment: | | | | | |
| --- | --- | --- | --- | --- | --- |
|  | not available at all | too few available | scarce | sufficient | I don’t know |
| Were there enough **mouth-nose-masks** available for you in March/April 2020? | □ | □ | □ | □ | □ |
| How do you estimate the availability of **mouth-nose-**  **masks** in the event of another  Pandemic wave? | □ | □ | □ | □ | □ |
| Were there enough **FFP2-masks** available for you in March/April 2020? | □ | □ | □ | □ | □ |
| How do you estimate the availability of **FFP2-**  **masks** in the event of another  Pandemic wave? | □ | □ | □ | □ | □ |
| Were there enough **protective gowns** available for you in March/April 2020? | □ | □ | □ | □ | □ |
| How do you estimate the availability of **protective gowns** in the event of another  Pandemic wave? | □ | □ | □ | □ | □ |
| Were there enough **protective gloves** available for you in March/April 2020? | □ | □ | □ | □ | □ |
| How do you estimate the availability of **protective gloves** in the event of another Pandemic wave? | □ | □ | □ | □ | □ |
| Was there enough **disinfectant** available for you in March/April 2020? | □ | □ | □ | □ | □ |
| How do you estimate the availability of **disinfectant** in the event of another Pandemic wave? | □ | □ | □ | □ | □ |
| Were there enough **face-shields** for you in March/April 2020? | □ | □ | □ | □ | □ |
| How do you estimate the availability of **face-shields** in the event of another Pandemic wave? | □ | □ | □ | □ | □ |
| Were there enough **protective goggles** for you in March/April 2020? | □ | □ | □ | □ | □ |
| How do you estimate the availability of **protective goggles** in the event of another Pandemic wave? | □ | □ | □ | □ | □ |

| 14. Do you inform yourself professionally about the Corona pandemic? | | |
| --- | --- | --- |
| □ yes | □ no | □ I don’t know |

| 14.1 If yes, which way do you obtain information about covid-19? **(multiple choice)** | | |
| --- | --- | --- |
| □ employer | □ public media | □ podcast |
| □ professional journal for HCAs | □ professional association | □ DEGAM guidelines |
| □ social media | □ other (please specify): |  |

| 15. Does your practice **software** include a quality management system (QM-system)? | | |
| --- | --- | --- |
| □ yes | □ no | □ I don’t know |

| 15.1 If yes, did the QM system of your practice software help you to cope with the Corona Pandemic? | | |
| --- | --- | --- |
| □ yes | □ no | □ I don’t know |

| 16. Are there other forms of support you would like in a **future Pandemic wave**? |
| --- |
| free text: |

| 17. In case of contact with covid-19-infected people, have you or personnel in your practice been quarantined? | |
| --- | --- |
| □ yes | □ no |

| 18. Has your practice had to be closed due to an infection of you or the staff? | |
| --- | --- |
| □ yes | □ no |

| 18.1 If yes, for how long? |
| --- |
| Days:____ |

| 19. Did the pandemic **in March/April** have an impact on your weekly working hours?  **(Multiple selection, please tick all that apply)** | | |
| --- | --- | --- |
| □ the weekly working hours were shorter than usual | □ the weekly working hours were longer than usual | □ I spend more time with my patients than usual |
| □ I spend less time with my patients than usual | □ I spend more time on organizational activities than usual | □ I spend less time on organizational activities than usual |
| □ no impact | □ other (please specify): | |

| 20. Does the pandemic **currently** have an impact on your weekly working hours?  **(Multiple selection, please tick all that apply)** | | |
| --- | --- | --- |
| □ the weekly working hours are shorter than usual | □ the weekly working hours are longer than usual | □ I spend more time with my patients than usual |
| □ I spend less time with my patients than usual | □ I spend more time on organizational activities than usual | □ I spend less time on organizational activities than usual |
| □ no impact | □ other (please specify) | |

| 21. Did the Corona Pandemic cause any difficulties for you in balancing family and career (e.g. due to a lack of childcare)? | | |
| --- | --- | --- |
| □ yes | □ no | □ sometimes |

| 22. Has short-time work* been introduced in your practice? (*temporary reduction of regular working hours with reduced salary due to a significant loss of work) | | |
| --- | --- | --- |
| □ yes | □ no | □ I don’t know |

| 23. Have you been affected by short-time work*? | | |
| --- | --- | --- |
| □ yes | □ no | □ partly |

| 24. Do you personally expect financial losses as a result of the Corona Pandemic compared to last year? | | |
| --- | --- | --- |
| □ yes | □ no | □ I don’t know |

| 25. Are you worried you could lose your job because of the Corona Pandemic? | | |
| --- | --- | --- |
| □ yes | □ no | □ I don’t know |

| 26. Did you feel psychologically burdened by the Pandemic in **March/April**? | | | | | |
| --- | --- | --- | --- | --- | --- |
| not at all | very little | a little | medium | strong | very strong |
| □ | □ | □ | □ | □ | □ |

| 27. Was there professional psychological support available for you in **March/April**? | | | | | |
| --- | --- | --- | --- | --- | --- |
| never | rarely | occasionally | often | always | no need |
| □ | □ | □ | □ | □ | □ |

| 28. Please rate your agreement or disagreement with the following statements: | | | | | |
| --- | --- | --- | --- | --- | --- |
|  | totally agree | tend to agree | indifferent | tend to disagree | totally disagree |
| I am exposed to an increased  risk of infection due to the Corona Pandemic | □ | □ | □ | □ | □ |
| Personally, I am afraid of getting sick with covid-19 | □ | □ | □ | □ | □ |
| I am afraid of dying in case of a covid-19 disease | □ | □ | □ | □ | □ |
| I am worried that my relatives could get seriously sick with covid-19 | □ | □ | □ | □ | □ |

| 29. Do you think that the Corona Pandemic will also lead to long-term changes in primary care/your daily work routine? | | |
| --- | --- | --- |
| □ yes | □ no | □ I don’t know |

| 29.1 If yes, are you worried about these changes? | | | | | |
| --- | --- | --- | --- | --- | --- |
| not at all | very little | a little | medium | strong | very strong |
| □ | □ | □ | □ | □ | □ |

| **Now some information about yourself:** |
| --- |

| 30. Gender | | |
| --- | --- | --- |
| □ male | □ female | □ diverse |

| 31. Age |
| --- |
| ___ years |

| 32. Do you belong to the risk group for a severe course of Covid-19 disease? | | |
| --- | --- | --- |
| □ yes | □ no | □ I don’t know |

| 33. Are there people in your household who belong to a risk group for Covid-19? | | |
| --- | --- | --- |
| □ yes | □ no | □ I don’t know |

| 34. location of the practice | |
| --- | --- |
| □ large city (>100.000 people) | □ small town (5.000 – 20.000 people) |
| □ medium city (>20.000-100.000 people) | □ rural location (<5.000 people) |
| □ other (please specify) | |

| 35. In which federal state is your practice located? | |
| --- | --- |
| □ Bavaria | □ Baden-Wuerttemberg |
| □ Schleswig-Holstein | □ Mecklenburg-Western Pomerania |

| 36. How long have you been active in your profession? (If you are unsure, please indicate approximate number of years) |
| --- |
| for____ years |

| 37. What is your position/role in the practice? | |
| --- | --- |
| □ Employee HCA | □ Trainee |
| □ Lead HCA | □ Other (please specify) |

| 38. What is the structure of the practice in which you work? (multiple choice) | |
| --- | --- |
| □ individual practice | □ single-site practice |
| □ group practice | □ multi-site practice (multi-settlement) |
| □ medical care center (MVZ) | |

| 38. How many physicians work in the practice? |
| --- |
| Number of physicians: ___ |

| 40. How many health care assistants work in the practice? |
| --- |
| Number of HCAs: ___ |

**Thank you for your support!**

**Supplementary material 5: Interview guide.**

Informing study participants about:

- Content and purpose of the study
- Procedure of the interview
- Recording of the interview on tape or electronically as well as subsequent transcription in accordance with applicable data protection regulations (recording will only begin afterwards)
- Voluntary participation in the study and that non-participation is not associated with any disadvantages, as well as revocation of consent is possible at any time.

| **Interview no.:** |  |
| --- | --- |
| **Date of the interview:** |  |
| **Duration of the interview:** |  |
| **Interviewer:** |  |

1. **Introduction**

| *[Before the interview: Sending/handing over and signing the consent form for participation in the study]*  *[Welcome]* I am a doctoral student at the Institute of General Medicine ..... I would like to thank you very much for your participation also on behalf of the institute directors in Würzburg and Kiel.  Within the anonymous questionnaire we tried to get an overview of the organizational, health-related, psychosocial and economic impact of the Corona pandemic on primary care. In this interview, we would like to learn more about how you experienced the Corona pandemic in your practice. I am interested in your personal experiences here.  Briefly, about the interview process: In the following, I will ask you a few questions about the above-mentioned complex of topics. After that - if you agree - general data about you and your practice will be collected.  Now a few notes on data protection:  *[Clarification about conditions of participation/data protection according to the participant information].*  Do you have any further questions?  I would now record the interview as explained. Do you agree to this?  *[Switch on device here]*  As mentioned earlier, the following will be about how you experienced the Corona pandemic in your practice. I'm going to ask you a few questions now. You are welcome to take as much time as you like in answering them and to tell me in detail what comes to your mind on the subject. Even if it seems unimportant to you, just tell me everything that comes to your mind. We are also interested in aspects that go beyond the questions answered in the questionnaire. |
| --- |

1. **Thematic questioning**

| **First question:** Regarding the Corona pandemic, is there a topic you would like to start with or that immediately comes to mind? |
| --- |

| **Theme 1: Health System**   - Perspective on the health care system in the Corona pandemic - Subjective assessment of the care situation - Subjective perception of deficits in the provision of care - Own considerations and impulses for improving care | |
| --- | --- |
| **Guiding question/narrative impulse**  What was your experience with health care during the pandemic/since the pandemic began? | **Concrete requests**  How do you describe the care provided by primary care physicians during the pandemic?  How do you assess outpatient specialist and inpatient care during the pandemic?  What other positive and negative developments in dealing with the pandemic have you observed in the healthcare system?  What are your wishes regarding the care of Covid-19 patients?  From your point of view, what are the biggest problems in dealing with the pandemic at the moment?  In your view, what urgently needs to be improved? |

| **Theme 2: Experience in practice in terms of staff/organization**   - Impact of the Corona pandemic on practice organization - Impact of the Corona pandemic on the staff - Adaptation strategies in practice | |
| --- | --- |
| **Guiding question/narrative impulse**  What has changed in your practice over the course of the pandemic? | **Concrete requests**  How do you deal with the pandemic in your practice?  How has the pandemic affected the organization of your practice?  How did your staff react to the pandemic?  What impact did the pandemic have on your work?  To what extent has the pandemic affected your practice financially?  What was your experience in providing patient care during home and house visits? |

| **Theme 3:** **Experiences in practice with regard to patients**   - Impact on own treatment approach - Experiences with patients/relatives with Covid-19 | |
| --- | --- |
| **Guiding question/narrative impulse**  Which impact did the Corona pandemic have on your patient care? | **Concrete requests**  To what extent has the treatment of non-Covid patients changed for you in the Corona pandemic?  What steps have you taken to care for your covid patients? |

| **Theme 4: Personal involvement/contact**   - Own coping strategies - Use of and access to external support measures - Adaptation strategies in the private environment - Dealing with the disease in the professional environment (Mutual support? Being left alone? Denial of a risk?) - Dealing with the disease in family and acquaintances (support, stigmatization of the person exposed to Covid-19?) | |
| --- | --- |
| **Guiding question/narrative impulse**  How did you personally deal with the situation? | **Concrete requests**  What impact did the pandemic have on your private life?  How did your private environment react to the pandemic?  Did people in your environment get ill with Covid-19?  What forms of support did you experience during the corona pandemic? E.g., emergency care, government assistance.  What helped you to cope with the pandemic?  What impact did the Corona pandemic have on your family life? |

| **Last question:** Thank you very much for your description, it is very interesting.  I have worked through my questions. From your point of view, are there still topics that seem important to you?  *[Switch off device here]* |
| --- |

1. **Personal data**

| Asking general data about you and your practice: As already discussed, this personal data is categorized into rough intervals, i.e. instead of recording your exact age, for example, we assign you to an age group. This prevents conclusions being drawn about individual participants and ensures that your data is anonymized.  *[Request for personal data - if not already ticked on the consent form].* |
| --- |

| **Personal data MFA (if not already filled in during the consent form)**  30. Gender  □ male □ female □ diverse  Age in years  ___ years  Do you belong to the risk group for a severe course of Covid-19 disease?  □ yes □ no □ I don’t know  Are there people in your household who belong to a risk group for Covid-19?  □ yes □ no □ I don’t know  Location of the practice  □ large city (>100.000 people) □ small town (5.000 – 20.000 people)  □ medium city (>20.000-100.000 people) □ rural location (<5.000 people)  □ other (please specify)  In which federal state is your practice located?  □ Bavaria □ Baden-Wuerttemberg  □ Schleswig-Holstein □ Mecklenburg-Western Pomerania  How long have you been active in your profession? (If you are unsure, please indicate approximate number of years)  for____ years  What is your position/role in the practice?  □ Employee HCA □ Trainee  □ Lead HCA □ Other (please specify)  What is the structure of the practice in which you work? (multiple choice)  □ individual practice □ single-site practice  □ group practice □ multi-site practice (multi-settlement)  □ medical care center (MVZ)  How many physicians work in the practice?  Number of physicians: ___  How many health care assistants work in the practice?  Number of HCAs: ___ |
| --- |

1. **Prospects and Goodbye**

| - Thank - Note questions and comments - Include contact information for information on study results including publications. - *[note mail address if applicable]* - *[Farewell]* |
| --- |

Supplementary material 6: Selection of stress-related items based on Winefield (2003).

| **Sources of Stress (Winefield, 2003)** | **Selected Survey Items** |
| --- | --- |
| **1) Patients as a sources of stress**  (e.g. patient care, patients diseases) | - Item 2: competence in caring COVID March/April - Item 3: competence in caring COVID-19 currently - Item 4: change of care non-COVID-19 |
| **2) Non-patient sources of stress**  (e.g. relations with co-workers, the juggling of emotional and time demands between work and family life (especially for women)) | - Item 21: work-life-balance and child care - Item 24: expected financial loss - Item 25: worry job losing - Item 28: risk of infection |
| **3) Organizational sources of stress**  (e.g. workload, paper work, responsibilities, lack of career path, decreasing professional autonomy, lack of support) | - Item 5: current patient volume - Item 10: support employer - Item 11: satisfaction government - Item 12: satisfaction employer - Item 13: Availability FFP2-masks march/April - Item 17: quarantine - Item 18: practice closing - Item 19: weekly working hours March/April - Item 20: weekly working hours currently - Item 22: short-time work practice - Item 23: short-time work individual |

**Supplementary material 7:**

**Supplementary Figure 3**. Code frameworks: a) Code framework of Ehlers-Mondorf et al. and b) reanalyzed code framework focusing stress experiences.

**Supplementary material 8: Triangulation Protocol.**

| **Stress experience of HCA** |
| --- |

|  | **Key Findings** | **S-QUAN** | **S-QUAL** | **I- QUAL** | **Triangulation** |
| --- | --- | --- | --- | --- | --- |
| 1 | Psychical burden was high among HCAs. | agree | no data | agree | silence |

| 2 | Especially in March/April HCAs reported feeling overburden. | agree | no data | agree | silence |
| --- | --- | --- | --- | --- | --- |
| 3 | HCAs reported a missing of offers for professional psychological support. | agree | no data | agree | silence |
| 4 | HCAs reported problems with compatibility of family and work, e.g. lack of childcare. | agree | agree | agree | agreement |
| **Patients as a source of stress** | | | | | |

|  | **Key Findings** | **S-QUAN** | **S-QUAL** | **I- QUAL** | **Triangulation** |
| --- | --- | --- | --- | --- | --- |

| 5 | HCAs reported lower experience of competence treating Covid-19 patients in March/April but over time it got better. | agree | no data | agree | silence |
| --- | --- | --- | --- | --- | --- |
| 6 | HCAs reported that health care of non-Covid-19 patients suffered harms. | agree | agree | agree | agreement |
| 7 | HCAs reported uncertainty with the new disease and wanted to be well informed. | agree | agree | agree | agreement |
| **Non-patient sources of stress** | | | | | |
|  | **Key Findings** | **S-QUAN** | **S-QUAL** | **I- QUAL** | **Triangulation** |

| 8 | Some HCAs reported fear of infection. | agree | no data | agree | silence |
| --- | --- | --- | --- | --- | --- |
| 9 | Most HCAs were afraid of infecting family members and themselves. | agree | no data | agree | silence |
| 10 | HCAs reported a wish for regular testing of medical personal. | no data | agree | agree | silence |
| 11 | Some HCAs reported worries about their financial situation. | agree | agree | agree | agreement |
| 12 | HCAs wished to be financially supported (e.g. bonus payment, salary). | no data | agree | agree | silence |
| 13 | HCAs reported difficulties in reconciling work and family life (e.g. unavailable childcare). | agree | agree | agree | agreement |
| 14 | HCAs reported the wish for support in availability of childcare. | no data | agree | agree | silence |

| **Organizational sources of stress** |
| --- |

|  | **Key Findings** | **S-QUAN** | **S-QUAL** | **I- QUAL** | **Triangulation** |
| --- | --- | --- | --- | --- | --- |

| 15 | HCAs reported changes in workload. | agree | no data | agree | silence |
| --- | --- | --- | --- | --- | --- |
| 16 | HCAs reported an increased workload due to administrative or organizational tasks and bureaucratic processes. | agree | agree | agree | agreement |
| 17 | Only a few HCAs reported that there was short-time work. | agree | agree | agree | agreement |
| 18 | HCAs reported problems with billing procedure of physician services. | no data | agree | agree | silence |
| 19 | HCAs reported support from their employer and good teamwork. | agree | no data | agree | silence |
| 20 | HCAs reported satisfaction with employers’ action regarding to the Corona pandemic. | agree | not data | agree | silence |
| 21 | HCAs reported low satisfaction with state government's handling of the Corona pandemic. | agree | agree | agree | agreement |
| 22 | HCAs reported frustration about measures to stop infections | no data | agree | agree | silence |
| 23 | HCAs reported frustration about information management | no data | agree | agree | silence |
| 24 | HCAs reported frustration about organization and structures. | no data | agree | agree | silence |
| 25 | HCAs reported a missing of contact persons and their reachability, e.g. at health department, health insurance, medical association. | no data | agree | agree | silence |
| 26 | HCAs reported poor supply of personal protective equipment in March/April, e.g. masks. | agree | agree | agree | agreement |
| 27 | HCAs reported a wish for relieving the burden on general practitioners. | no data | agree | agree | silence |
| 28 | HCAs reported a wish for (re)opening of swab test centers. | no data | agree | agree | silence |
| 29 | HCAs reported a wish for covid-19 specific offers, e.g. corona centers, infection practices, infection consultation, infection consultation. | no data | agree | agree | silence |
| 30 | HCAs reported a lack of recognition and appreciation of their work by the government and society. | no data | agree | agree | silence |

|  | S-QUAN: quantitative data of the survey; S-QUAL: qualitative data of open-end survey questions;  I-QUAL: qualitative data of interviews |
| --- | --- |

**Supplementary material 9: Sociodemographic data of the qualitative interview participants.**

| **Age (Years)** | |  | **Gender** | |
| --- | --- | --- | --- | --- |
| 18-30 | 1 (3%) |  | Female | 33 (97%) |
| 31-40 | 12 (35%) |  |  |  |
| 41-50 | 8 (24%) |  | Male | 1 (3%) |
| 51-60 | 13 (38%) |  |  |  |
| **Years of profession** | |  | **Position in practice** | |
| < 5 | 3 (9%) |  | HCA trainee | 1 (3%) |
| 5-14 | 10 (29%) |  |  |  |
| 15-24 | 8 (24%) |  | Employee HCA | 31 (91%) |
| 25-34 | 4 (12%) |  |  |  |
| 35-44 | 9 (26%) |  | Other | 2 (6%) |
| > 55 | 0 (0%) |  |  |  |
| **COVID-19 risk group** | |  | **Risk group in household** | |
| Yes | 9 (26%) |  | Yes | 16 (47%) |
| No | 25 (74%) |  | No | 18 (53%) |
| **Number of physicians** | |  | **Number of health care assistants** | |
| 1 | 14 (41%) |  | 1 | 0 (0%) |
| 2 | 12 (35%) |  | 2 | 13 (38%) |
| 3 | 4 (12%) |  | 3 | 3 (9%) |
| 4 | 0 (0%) |  | 4 | 10 (29%) |
| 5 or more | 4 (12%) |  | 5 or more | 8 (24%) |
| **Location of the practice (inhabitants)** | |  | **Federal state of practice** | |
| Rural (<5000) | 14 (41%) |  | Baden-Wuerttemberg | 9 (27%) |
| Small town (5,000-20,000) | 4 (12%) |  | Bavaria | 7 (21%) |
| City (>20,000-100,000) | 11 (32%) |  | Mecklenburg-Western Pomerania | 7 (21%) |
| Large city (>100,000) | 5 (15%) |  | Schleswig-Holstein | 11 (32%) |
| **Practice structure** | |  |  |  |
| Individual practice | 19 (56%) |  |  |  |
| Joint practice | 13 (38%) |  |  |  |
| Medical care center | 0 (0%) |  |  |  |
| Practice with several locations (multi-settlement) | 2 (6%) |  |  |  |
| Practice with single location | 0 (0%) |  |  |  |

**Supplementary material 9: Feeling of overburden in daily practice.**

| **Feeling of overburden in daily practice** | **March/April 2020 (N=1251; m=23)*** | **Time of survey Aug-Dec 2020 (N=1245; m=29)*** |
| --- | --- | --- |
| Not at all | 113 (9.0%) | 172 (13.8%) |
| Very low | 96 (7.7%) | 190 (15.3%) |
| Low | 183 (14.6%) | **294 (23.6%)** |
| Medium | **285 (22.8%)** | 364 (29.2%) |
| High | 356 (28.5%) | 150 (12.0%) |
| Very high | 218 (17.4%) | 75 (6.0%) |
| Vargha-Delaney A | A=34.5% (32–36) p<0.001 | |
| The missing values (m) were not included in the percentage calculation.  *The median levels of ordinal and continuous scale variables are highlighted in bold numbers | | |

**Supplementary material 10: Additional verbatim quotes.**

|  | ***Stress experience of HCAs*** |
| --- | --- |
| **VS1** | *"[...] I am also a high-risk patient, I have asthma, chronic and allergic asthma. I'm always a bit worried, so every day is a ride on a razor's blade for me. My husband has Parkinson's disease and high blood pressure. At the beginning of the pandemic, I was still looking after my mother-in-law, who lived next door to us. That was very difficult because she was a bit demented. She also died in the meantime. And it was all a very bad situation.” (No. 9, pos. 50)* |
| **VS2** | *"[...] as soon as the Corona started with this lockdown, I totally got the rheumatic attacks." (No. 1, pos. 16)* |
| **VS3** | *"Perhaps one or the other would have needed more options to talk. […] opportunities of where such people go with their worries, with their needs. It is not necessarily always the boss who can be the contact person. That is also a practical conflict.“ (No. 2, pos. 44-46)* |
|  | ***Patients as a source of stress*** |
| **VS4** | *"Now what to speak, what is a worried patient and what is a sick patient, who really needs help?" (No. 2, pos 8)* |
| **VS5** | *"The HCA must decide: What shall we do with such a patient in the first place? Do you have to call them in? Do you have to transfer them? And if yes, where?“ (No. 15, pos. 44)* |
| **VS6** | *"[…] so in the spring we were actually overwhelmed because there was simply too little knowledge. “ (No. 21, pos. 4)* |
| **VS7** | *“Of course, masks are obligatory, that's clear, and it wasn't very well accepted at the beginning, which meant for us as sisters that we discussed a lot or were forced to discuss a lot, and sometimes we had to become strict.” (No. 26, pos. 22)* |
| **VS8** | *"That's more now, as the numbers are getting higher now, of course we are getting more panicky patients, need to make more phone calls because patients just panic and of course they call us, and we have to reassure them, explain a little bit and they were, are now also sometimes afraid to come to the surgery. “ (No. 20, pos. 16)* |
| **VS9** | *"[…] Also because many patients are more dissatisfied, more aggressive, which I already said, you have to be scolded why things are not moving forward. People have to stand outside in the rain in the cold. But as I said, they are all just people and we can't do more than work. Of course, we also have patients who really praise us and say: "Wow, that's great how you do everything here and how you handle it. You have both encouragement, but also patients who are sometimes, I would say, indignant.” (No. 7, pos. 12)* |
| **VS10** | *“B: Many [patients] just avoid coming to the practice. Which can of course be unfavourable for the patients when, for example, an ECG should be done because of certain pains and they just don't come.*  *I: Have you really experienced that someone was harmed because they didn't come because of the ECG, for example?*  *B: No, I wouldn't say that, but recently, for example, a patient was brought to the hospital at night with a suspected heart attack and I could imagine that if everything had been normal, he might have come to the practice before.” (No. 23, pos. 8)* |
| **VS11** | *"We've also had bad experiences where some people have died because they actually didn't get in touch. But thanks God, very, very few.“ (No. 12, pos. 24)* |
|  | ***Non-patient sources of stress*** |
| **VS12** | *"And yet when I think that some now here in the area, yes some have closed, actually closed their surgeries, out of concerns about the Corona.“ (No. 2, pos. 44)* |
| **VS13** | *“Cases where the doctor was not sure, she sent them to the smear centre. But more actually because of the fact that the doctor was really afraid that our practice would be closed. […] Because someone is infected, as we have it right now with a partner practice with whom we always alternate with holiday replacement. […]” (No.26, pos. 49)* |
| **VS14** | *“Well, it's an enormous pressure, an enormous responsibility that rests on us, and on top of that, of course, there's the whole private life. Corona not only worries us at work in the practice, but also in our private life. We are afraid of what we will take home with us. One has a sick grandmother or a sick husband or many small children. You just worry a lot.” (No. 25, pos. 16)* |
| **VS15** | *"She then went on sick leave because she said she was so worried about the situation and in such a fear that it was so close to her and got under her skin that she then just couldn't do it. “ (No. 2, pos. 10)* |
| **VS16** | *"And see that I protect my parents. My daughter hasn't been there since then either, and my husband not anyway. If anyone only I went there. And we made sure that we all didn't have any private parties, so they were all called off.“ (No. 9, pos. 49)* |
| **VS17** | *“You can't go to the beach anymore, you can't do sports, you can't go for walks by the sea, now you have to go into the forest […]. So you have to change. But that doesn't mean / you have to change, but you are already more organized with the meetings of friends, they are phoned. So it works differently.” (No. 19, pos. 97)* |
| **VS18** | *“Yes, I think that compensation due to leisure time is so lacking. It's actually the case that you work the whole day, that's okay. And it was always difficult in the winter quarter in the practice, you always had more stress, but then you knew: Ok, something is planned for the weekend. I'm meeting someone, I don't know, we've invited someone. And it just stopped now. And over the summer you could compensated it a little bit by at least meeting outside. The weather was good, we were out, that's fine, but now, slowly, when it's getting darker outside again and now, of course, with the new restrictions on contact, it's really affecting our mood. At this time, the mood is really in the dumps.” (No. 5, pos. 74)* |
| **VS19** | *"[…] what I notice, in my apartment building there is an elevator and if I stand in the elevator with a mask on, no one gets in next to me nevertheless. They always wait.“ (No. 21, pos. 52)* |
| **VS20** | *"[…] then my mother, who is completely against everything. I also had a bit of stress with her because of the whole story.“ (No. 1, pos. 42)* |
| **VS21** | *"There were times […] we had reduced weekly working hours, because simply […] no people came and it was a hard time, I have to say quite honestly.“ (No.1, pos. 16)* |
| **VS22** | *"So it was a bit difficult, we had to take turns taking leave or the grandma or the grandpa. It was the early days, when we didn't know anything. And then giving our child to the grandma and grandpa, even though we didn't know whether we or our child were somehow positive.“ (No. 25, pos. 40)* |
| **VS23** | *“Then I came home, then the children were there. They don't understand the homework, they have to do this and then I sat there with them until evening and did homework. So it was really - I have to say quite honestly - I'm glad that school has now started again. And I hope it stays that way, because then you have a bit of a rest again. You have a bit of time again. You come home from work and can have a quick coffee before everyone [the children] is jumping around you again.” (No. 1, pos. 16)* |
| **VS24** | *"Well, yes, I also have a small child who is in the kindergarten. Then the time when the kindergartens were closed was also a huge drama at the beginning. Of course, I was always afraid at the beginning that I would spread something to my daughter or to my grandmother, who is actually very ill. But at the beginning, in March and April, contacts were limited and it was difficult to manage child and work, because at the beginning it wasn't under emergency care.” (No. 25, pos. 38)* |
| **VS25** | *"And she [the colleague] has now resigned. She has two elementary school children […] that has all become too much for her now and she has suddenly resigned as of 1 December." (No. 23, pos. 32)* |
| **VS26** | *"We all have our jobs, we had to go and the little one had to stay at home. […] My colleagues were completely supportive with me and bearing the brunt.“ (No. 17, pos. 26)* |
| **VS27** | *“For a short time it was quite difficult. I have to be honest that every one of us reached the limit. Thank God, we have a great team, where almost no one was sick because they were overworked, or thank God, they didn't get sick, good luck. But of course we were often nagging and grumbling at each other, and we were right, because we had to remember that there was a lot of pressure, a lot of responsibility on us.” (No. 25, pos. 16)* |
|  | ***Organizational stress sources*** |
| **VS28** | *“It is more chaotic. It is simply more chaotic because the numbers have always changed, the procedures have changed every time. New forms come, other forms go. It is chaos.” (No.19, pos. 34)* |
| **VS29** | *"[…] we had no protective clothing available, we had no FFP2 masks. We actually had nothing at all.“ (No. 21, pos. 4)* |
| **VS30** | *“Yes, there were three situations where there was a shortage, where the delivery didn't come or there was sometimes very, very little, where the bosses sometimes managed with too fewer staff, so that we could save the protective gowns until the next delivery came. […]” (No. 25, pos. 26)* |
| **VS31** | *“And I remember a specialist surgeon who even closed his practice on two Fridays. And he and his HCAs sewed these everyday masks themselves because they didn't have anything left. That was even in the press, so I found that a bit alarming.” (No. 9, pos. 16)* |
| **VS32** | *"And we actually work the whole day actually only with mouth guards, are actually already dead tired at noon […].“ (No.4, pos. 14)* |
| **VS33** | *“So on the issue of masks, I can't agree with my colleague at all, because that's the way it is now, we use the mask, that's good for us. It's just going on for a while, we have to get through it. I'm accepting it, if I stay healthy. And consequently my parents, my old ones, stay healthy.” (No. 15, pos. 110)* |
| **VS34** | *“Well, I have to say that the time was really stressful for me and also, as I said, something changed every day, every day there was another letter from the KV [german: Kassenärztliche Vereinigung; Association of Statutory Health Insurance Physicians] where you had to reorganise yourself again. So we did so much organizational work. I'm only there for four hours in the morning, and in those four hours I just studied e-mails, which is now new again, created new things in the computer, which was then different again the next day.” (No. 1, pos. 16)* |
| **VS35** | *“It's kind of annoying because you don't get much support from the departments and government agencies.” (No. 7, pos. 2)* |
| **VS36** | *“But despite everything, there are always reports about nurses and care staff and everything, and I think that these GPs are mentioned too rarely. And I would simply wish for the future that GPs simply gets more encouragement and just somewhere a little thank you, something like that. For what you actually do here every day. Foremost, we as GPs are the first point of contact for patients. And I think that is valued too little. I think that's such a shame, it makes me sad.” (No. 7, pos. 60)* |
| **VS37** | *"Health departments were not available. We were always told, "Busy," or, "Contact your family doctor." We felt downright left all alone.“ (No. 16, pos. 4)* |
| **VS38** | *“And I searched […] everywhere on the internet, to see if there was any training, […]. But there wasn't, and there still isn't.” (No. 26, pos. 4)* |
| **VS39** | *"So I just think the enforcement of the measures itself would be easy, also in the public, with penalties that are higher, so it really hurts people when they sort of go against the bans.“ (No. 18, pos. 72)* |
|  | ***Resources*** |
| **VS40** | *"This year there was no traveling, we did not do that. We then made day trips here ourselves[…].“ (No. 6, pos. 58)* |
| **VS41** | *"[…]the bond with the family is stronger now […].“ (No. 15, pos. 110)* |
| **VS42** | *"[…] I'm trying to see the positive side of this all […]. I hope that my positive attitude will help me further.“ (No. 24, pos. 60)* |
| **VS43** | *"The more I know, the more confident I feel." (No. 16, pos. 24)* |

**Supplementary material 11: Additional verbatim comments.**

|  | ***Patients stress sources*** |
| --- | --- |
| **VC1** | Patients should be *“reasonable”, “less stressful”, “patently”, “not so bad”***.** |
| **VC2** | There was the wish for *“sympathy of patients and relatives”.* |
| **VC3** | *“Support from the health departments so that patients can be well informed and don`t have to turn to us helplessly because the departments are overloaded.”* |
| **VC4** | *“Structured information material for patients”* |
| **VC5** | *“Communication of the real risk of disease. Avoidance of panic and horror messages. Information on health protection measures.”* |
|  | ***Non-patients stress sources*** |
| **VC6** | *“Financial compensation for HCAs! We are also system relevant and not mentioned anywhere!”* |
| **VC7** | *“Keep COVID-19 out of practices as much as possible”* |
| **VC8** | *“That medical staff are also tested. Medical practices are left alone with this.”* |
|  | **Organizational stress sources** |
| **VC9** | *“We currently buy gloves, masks etc. at far overpriced prices (3-4 times the normal price) in order to protect ourselves.”* |
| **VC10** | *“More information, we felt very uninformed and helpless at the beginning. We only had information from the news and were supposed to calm patients down. We were told it was our own fault if we didn't have protective equipment in stock.”* |
| **VC11** | *“Clear information and uniform, well thought-out regulations that are easy to implement for a practice.”* |
| **VC12** | *“Better information on the "bureaucratic aspects", information sheets on billing procedure and coding were often incomplete, ambiguous, not pertinent for GPs; frequent changes [of information] and we had to tediously collect information on our own.”* |
| **VC13** | *“That we are given the same attention and support as was given to the hospital staff. After all, GPs are the first point of contact for infectious patients or the fears and worries associated with the pandemic.”* |
| **VC14** | *“Reopening of the test station by the coordinating doctor to avoid contact between infected or suspicious patients and non-infected patients.”* |

**Supplementary material 12: Worries in daily practice.**

| **What are you currently concerned about in terms of practice? (N=1274)*** | |
| --- | --- |
| Overlooking COVID-19 disease in patients | 460 (36.1%) |
| I am not concerned about COVID-19 | 132 (10.4%) |
| Contradictory or too little Information on COVID-19 | 638 (50.1%) |
| Patients being infected by the practice team | 441 (34.6%) |
| That employees could become infected | 863 (67.7%) |
| Suffering financial losses | 410 (32.2%) |
| **Worries about losing job due to the pandemic (N= 1252; m=22)** | |
| Yes | 73 (6.2%) |
| No | 1108 (93.8%) |
| I don’t know | 71 |
| The missing values (m) and answer “I don’t know” were not included in the percentage calculation.  *Multiple choice possible | |

**Supplementary material 13: Impact of the pandemic on workload and time spending.**

| **Impact of the pandemic on (N=1274)*…** | | | |  |
| --- | --- | --- | --- | --- |
| **… weekly working hours** | **March/April 2020** | | **Time of the survey Aug-Dec 2020** |  |
| Shorter than usual | 468 (36.7%) | | 57 (4.5%) |  |
| Similar to | **593 (46.6%)** | | **937 (73.5%)** |  |
| Longer than usual | 213 (16.7%) | | 280 (22.0%) |  |
| Longer than in Mar-Apr | A=65.0% (63–67) p<0.001 | | |  |
| **… time spent on organization** | **March/April 2020** | | **Time of the survey Aug-Dec 2020** |  |
| More time than usual | **727 (57.1%)** | | 581 (45.6%) |  |
| Less time than usual | 59 (4.6%) | | 49 (3.8%) |  |
| No impact | 488 (38.3%) | | **644 (50.6%)** |  |
| Less than in Mar-Apr | A=45.2% (43–46) p<0.001 | | |  |
| **… time spent with patients** | **March/April 2020** | | **Time of the survey Aug-Dec 2020** |  |
| More time than usual | 157 (12.3%) | | 146 (11.6%) |  |
| Less time than usual | 339 (26.6%) | | 135 (10.6%) |  |
| No impact | **778 (61.1%)** | | **993 (77.8%)** |  |
| More time than in Mar-Apr | A=56.4% (55–58) p<0.001 | | |  |
| *The median levels of ordinal and continuous scale variables are highlighted in outlined numbers**.** | | | |  |
| **Patient volume in practice at the time of the survey (N=1246; m=28)*** | | | | |
| Higher than before | |  | 208 (16.8%) | |
| Comparable | |  | **689 (55.7%)** | |
| Lower than before | |  | 339 (27.4%) | |
| I do not know | |  | 10 | |
| The missing values (m) and the answer “I don`t know” were not included in the percentage calculation.  *The median levels of ordinal and continuous scale variables are highlighted in outlined numbers. | | | | |

**Supplementary material 14: Satisfaction with your employers’ actions.**

| **Satisfaction with your employer's actions (N=1246; m=28)** | | |
| --- | --- | --- |
| Very satisfied |  | 517 (41.5%) |
| Satisfied |  | **492 (39.5%)** |
| Medium |  | 172 (13.8%) |
| Dissatisfied |  | 53 (4.3%) |
| Very dissatisfied |  | 12 (1.0%) |
| The missing values (m) were not included in the percentage calculation. | |  |

**Supplementary material 15: Short-time work and FFP2 masks.**

| **Have reduced weekly working hours** been introduced in your practice? (N=1251; m=23)*** | | |
| --- | --- | --- |
| Yes | 162 (13.0%) | |
| No | **1085 (87.0%)** | |
| I do not know | 4 | |
| **Did your practice have to be closed due to infection of you or another member of staff? (N=1241; m=33)*** | | |
| Yes | 75 (6.0%) | |
| No | **1166 (94.0%)** | |
| **If yes, for how long? (days)** | | |
| Minimum | | 1 |
| Mean | | 10.8 |
| Median | | **10** |
| Maximum | | 115 |
| **Were there enough FFP masks available for you in March/April 2020? (N=1253; m=21)*** | | |
| Not available at all | | 369 (29.6%) |
| Too little available | | **479 (38.4%)** |
| Scarcely available | | 241 (19.3%) |
| Sufficiently available | | 159 (12.7%) |
| I do not know | | 5 |
| The missing values (m) and the answer “I don`t know” were not included in the percentage calculation.  *The median levels of ordinal and continuous scale variables are highlighted in outlined numbers  **Short-time work: temporary reduction of regular working hours with reduced salary due to a significant loss of work | | |

**Supplementary material 16: Risk of infection.**

|  | **Agree completely** | **Rather**  **agree** | **Indiff**  **-erent** | **Rather disagree** | **Disagree completely** |
| --- | --- | --- | --- | --- | --- |
| **Exposed to an increased risk**  **of infection (N=1255; m=19)*** | **792 (63.1%)** | 273 (21.8%) | 54 (4.3%) | 76 (6.1%) | 60 (4.8%) |
| **Afraid of getting sick with**  **COVID-19 (N=1254; m=20)*** | 196 (15.6%) | 289 (23.0%) | **224 (17.9%)** | 375 (30.0%) | 170 (13.6%) |
| **Afraid that “I will die if I am**  **getting sick” (N=1253; m=21)*** | 105 (8.4%) | 108 (8.6%) | 186 (14.8%) | **458 (36.6%)** | 396 (31.6%) |
| **Afraid that relatives could**  **get severely sick with**  **COVID-19 (N=1254; m=20)*** | 477 (38.0%) | **414 (33.0%)** | 143 (11.4%) | 155 (12.4%) | 65 (5.2%) |
| The missing values (m) were not included in the percentage calculation.  *The median levels are highlighted in boldface numbers. | | | | | |

**Supplementary material 17: Resources and coping strategies.**

| **Summary of resources and coping strategies (Interview QUAL)** |
| --- |
| - **problem focused strategies**   - searching for information (VS43, VS38)   - organizational skills for permanent adaption to new conditions and measurements (VS34)   - recreation of leisure time (e.g. planning day trips instead of vacations) (VS 18,40)   - improved team work, working all together (VS26, 27) - **emotional focused strategies**   - accepting and allowing negative feelings (VS42)   - family and friends (VS41) - **attributional focused strategies (VS42)**   - reattribution of the situations   - positive attitude |
| **Example Verbatims**  **VS43:** "The more I know, the more confident I feel." (No. 16, pos. 24)  **VS40:** *"This year there was no traveling, we did not do that. We then made day trips here ourselves[…].“ (No. 6, pos. 58)*  **VS41:** *"[…]the bond with the family is stronger at the time now […].“ (No. 15, pos. 110)*  **VS42:** *"[…] I'm trying to see the positive side of all this […]. I hope that my positive attitude will help me further.“ (No. 24, pos. 60)* |

**Literature**

1. Bohlken J, Schömig F, Seehagen T, Köhler S, Gehring K, Roth-Sackenheim C, et al. Erfahrungen und Belastungserleben niedergelassener Neurologen und Psychiater während der COVID-19-Pandemie. Psychiat Prax. 2020;47(04):214–7.

2. Schilling J, Tolksdorf K, Marquis A, Faber M, Pfoch T, Buda S, et al. Die verschiedenen Phasen der COVID-19-Pandemie in Deutschland: Eine deskriptive Analyse von Januar 2020 bis Februar 2021. Bundesgesundheitsbl. 2021 Sep;64(9):1093–106.
