# Supplementary figures and images for: Stress experiences of healthcare assistants in family practice at the onset of the COVID-19 pandemic: a mixed methods study
Source: Front Public Health. 2023 Sep 4;11:1238144. doi: 10.3389/fpubh.2023.1238144 (PMC10507357; doi:10.3389/fpubh.2023.1238144)

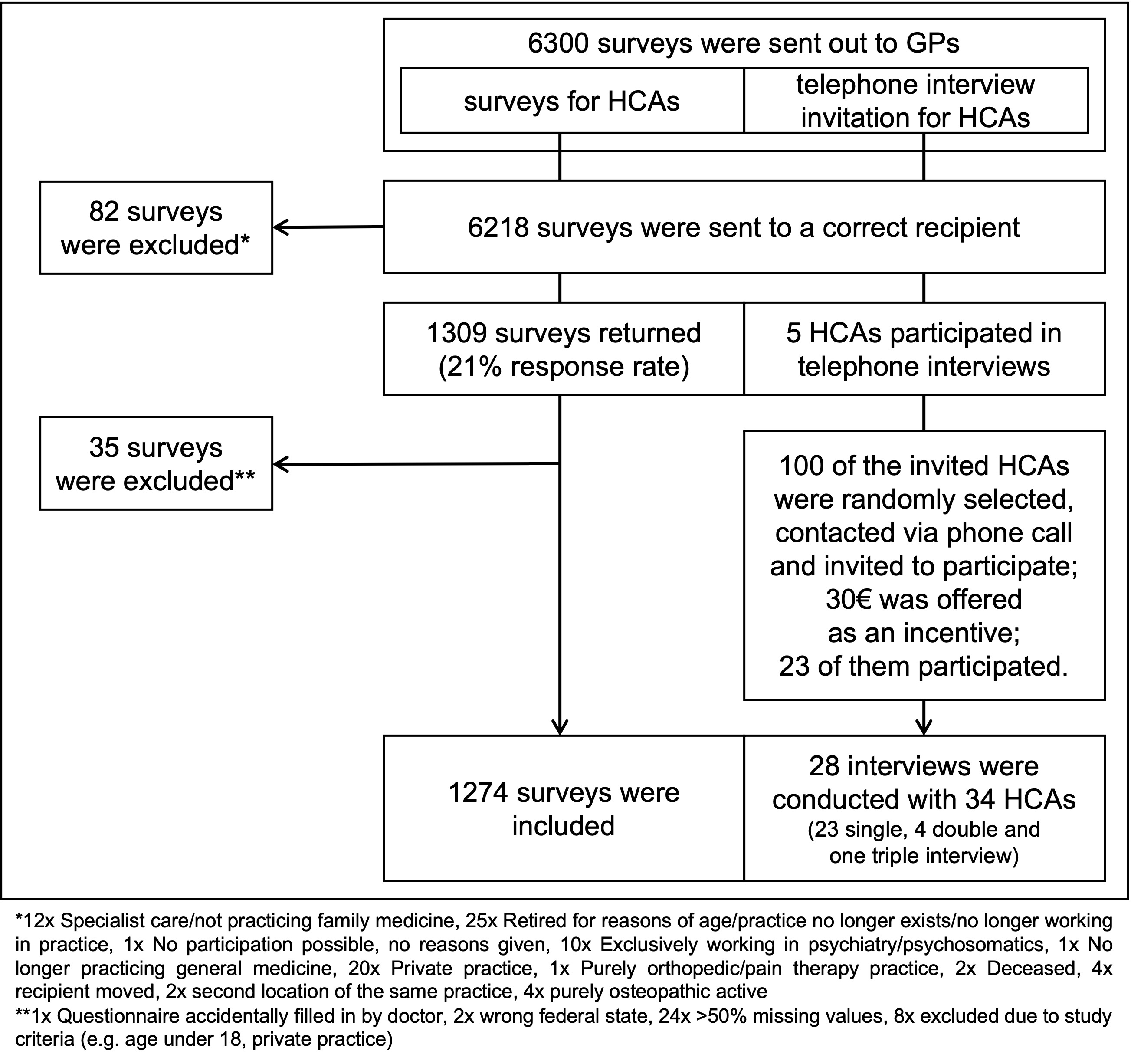

Supplement: Supplementary file 2 [file Image_1.JPEG]

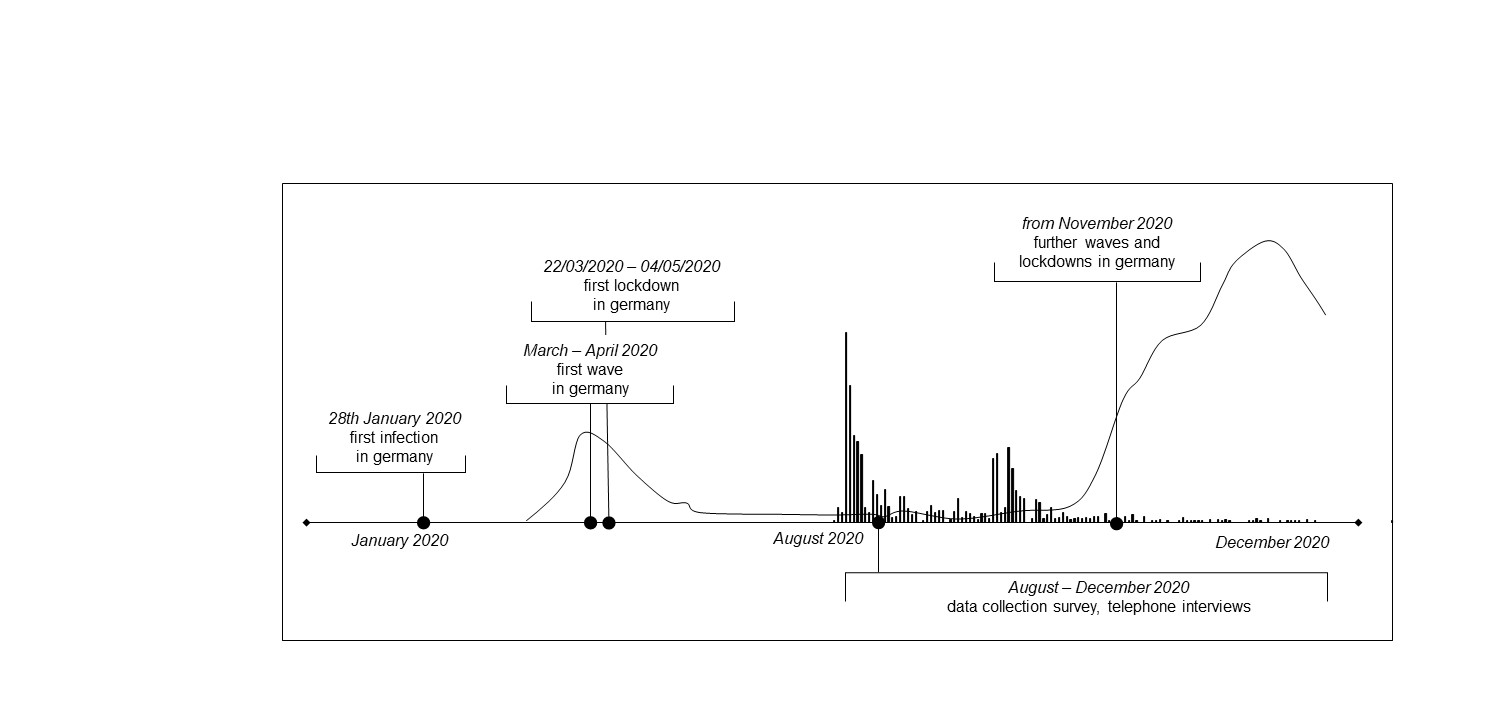

Supplement: Supplementary file 3 [file Image_2.jpg]

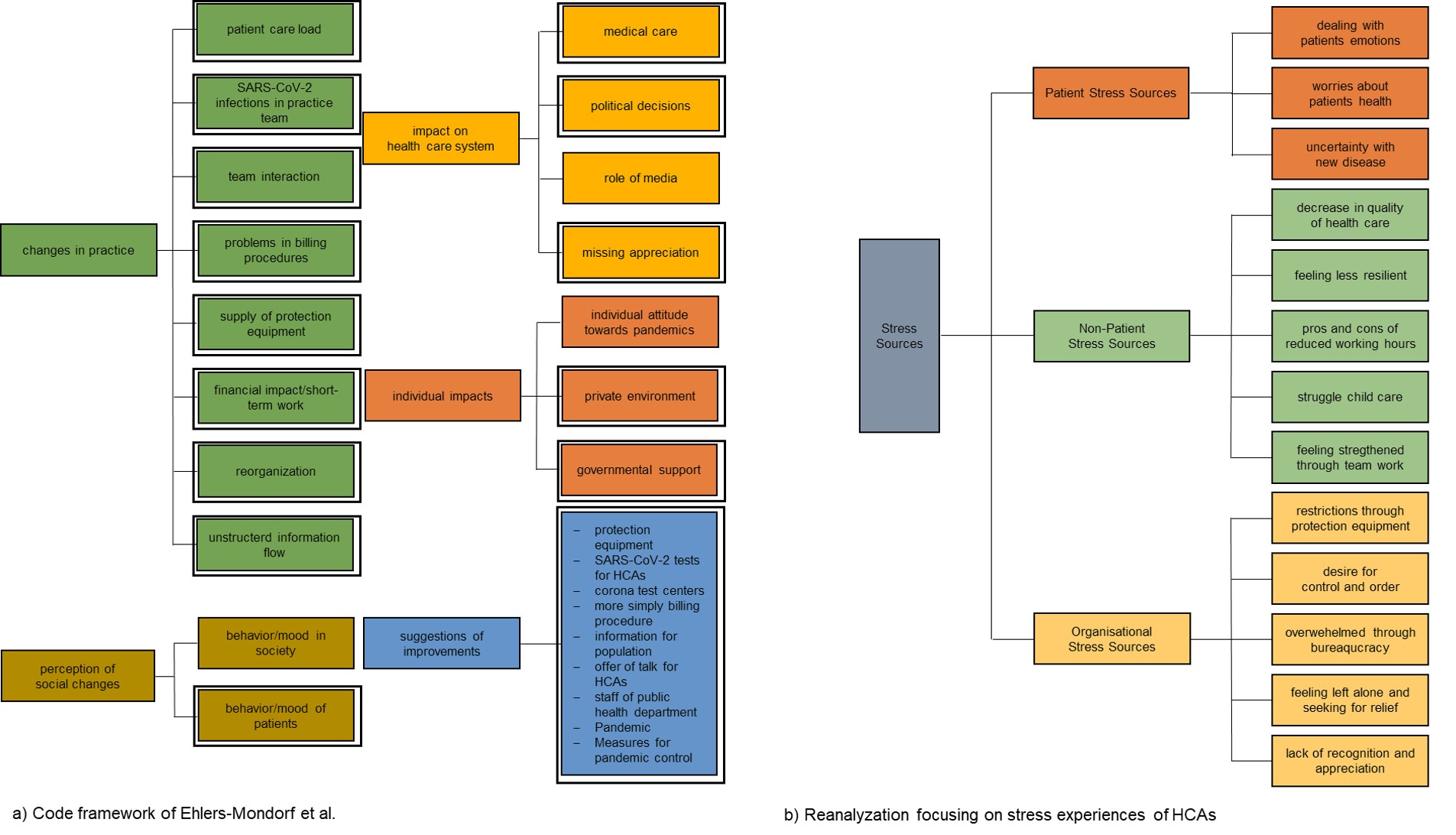

Supplement: Supplementary file 4 [file Image_3.jpeg]
